# Real-time monitoring of newly acidified organelles during autophagy enabled by reaction-based BODIPY dyes

**DOI:** 10.1038/s42003-019-0682-1

**Published:** 2019-11-28

**Authors:** Hanzhuang Liu, Wenting Song, Delia Gröninger, Lei Zhang, Yinghong Lu, Kin Shing Chan, Zhikuan Zhou, Knut Rurack, Zhen Shen

**Affiliations:** 1 State Key Laboratory of Coordination Chemistry, School of Chemistry and Chemical Engineering, Nanjing University, Nanjing, 210046 China; 2 Chemical and Optical Sensing Division, Bundesanstalt für Materialforschung und -prüfung (BAM), Richard-Willstätter-Str. 11, 12489 Berlin, Germany; 3 School of Chemical Engineering, Nanjing University of Science and Technology, Nanjing, 225600 China; 4 Department of Chemistry, The Chinese University of Hong Kong, Hong Kong, China

**Keywords:** Cellular imaging, Chemical modification, Fluorescent dyes, Autophagy

## Abstract

Real-time monitoring of newly acidified organelles during autophagy in living cells is highly desirable for a better understanding of intracellular degradative processes. Herein, we describe a reaction-based boron dipyrromethene (BODIPY) dye containing strongly electron-withdrawing diethyl 2-cyanoacrylate groups at the α-positions. The probe exhibits intense red fluorescence in acidic organelles or the acidified cytosol while exhibiting negligible fluorescence in other regions of the cell. The underlying mechanism is a nucleophilic reaction at the central *meso*-carbon of the indacene core, resulting in the loss of π-conjugation entailed by dramatic spectroscopic changes of more than 200 nm between its colorless, non-fluorescent leuco-BODIPY form and its red and brightly emitting form. The reversible transformation between red fluorescent BODIPY and leuco-BODIPY along with negligible cytotoxicity qualifies such dyes for rapid and direct intracellular lysosome imaging and cytosolic acidosis detection simultaneously without any washing step, enabling the real-time monitoring of newly acidified organelles during autophagy.

## Abstract (web summary)

Hanzhuang Liu et al. present new BODIPY dyes that undergo a reversible pH-dependent switch upon acidification from the colorless leuco-form to red fluorescent BODIPY. They use these probes to directly image organelle acidification associated with autophagy in real time.

## Introduction

In recent years, reaction-based fluorescence probes have attracted increasing attention by virtue of their superior selectivity and sensitivity^1–3^. A versatile strategy for the design of reaction-based probes is the ″pro-chromophore″ approach^4^, where a weakly (ideally zero) fluorescent pro-chromophore is transformed into its highly fluorescent parent dye through reaction with the target analyte. If this ″lighting up″ of the fluorescence is accompanied by a color change from colorless to a visible color and if the reaction itself is reversible, such a dye system belongs to the perhaps oldest class of responsive dyes: dyes that can be switched reversibly between a colorless leuco form and a colored all-π-conjugated form^5^. The essence of this strategy is a reaction at the core of the chromophore, resulting in the restoration (or loss) of π-conjugation^6–10^, rather than an analyte binding to a peripheral substituent on the dye. The latter most often only changes the charge distribution within the dye′s π-system, resulting in an analyte-induced modulation of a photophysical process such as an intramolecular charge transfer (ICT), a photoinduced electron transfer (PET) or Förster resonance energy transfer (FRET)^11^. Compared to ICT, PET or FRET, the transformation between a π-conjugated and a non-π-conjugated state generally offers dramatic spectral changes, minimal background and high output signals. Along this strategy, especially dyes based on the rhodamine skeleton have led to a variety of excellent fluorescent probes for super-resolution imaging^12^ and the detection of biologically relevant analytes^8,13–15^. In contrast, BODIPY (boron dipyrromethene) dyes, although having become virtually as popular as rhodamines because of their excellent spectroscopic properties including intense absorption and fluorescence, have only scarcely been developed as reactive probes undergoing attack of their core, most likely because of their generally accepted chemical robustness^16–19^. In 2015, Unaleroglu reported the first reversible fading phenomenon upon addition of base to an α-bis(methoxycarbonyl)ethenyl BODIPY solution, yet did not provide a mechanistic explanation for the changes happening in the chromophore^20^. Very recently, Yang reported the first reversible/quasi-reversible transformation of two *meso*-unsubstituted BODIPYs into a colorless dimer. They ascribed the unusual dimerization, which also proceeded in the presence of base, to dimerization of the BODIPY core and used this chemistry for formaldehyde detection and temperature measurement^10^. Making a reversible reaction at the core of a dye compatible with biological applications in aqueous media, however, is still an extremely important and challenging task^12–14^.

Acidified organelles are closely involved in autophagy processes, which themselves constitute a complex interplay of different metabolic pathways^21–24^. For example, autophagosomes can fuse with organelles of the endocytic pathway (early/late endosomes, multivesicular bodies), generating amphisomes that are more acidic than the autophagosomes. At the same time, as endosomes mature along an endocytic pathway they become commonly more acidic (pH ~5.0–5.5 for late vs. pH ~6.0–6.8 for early endosomes)^25,26^. Real-time imaging of the acidification of organelles is thus of great significance for a more comprehensive understanding of autophagy, which is closely associated with neurodegenerative diseases of aging, drug resistance in cancer treatment and tumor suppression^27,28^. Numerous molecular probes for long-lived acidic organelles such as lysosomes have been reported^29–31^. The general strategy commonly followed is to design probes that accumulate in such organelles. However, these probes usually do not allow to trace the actual formation of newly acidified organelles. Their identification and characterization mainly rely on electron microscopy. Electron microscopy, however, requires dead cells that have been chemically fixed. Live-cell imaging may be achieved by fluorescence microscopy by transfection of endosomal-specific protein genes. However, these methods require comparatively lengthy protocols and complicated operation steps while suffering from low efficiency. Consequently, they are mainly employed for the imaging of late endosomes. As an alternative, nanoprobes, especially ultrasensitive pH nanoprobes, can play an important role in the study of the mechanistic understanding of endocytosis and the intracellular trafficking of nanoparticles^32–35^, but ineffective intracellular delivery issues have largely hampered their application for the labeling of late endosomes and other acidified organelles during autophagy pathways. Small-molecule optical probes are more promising here because they are often biocompatible and frequently intrinsically membrane-permeable. Suitable probes for acidic organelles usually consist of a masked fluorophore with a lipophilic, weakly basic moiety that renders the probe acidophilic and promotes their accumulation in acidic vesicles upon protonation^29^. However, as mentioned above, this strategy does not apply to newly acidified organelles. When cells are incubated with molecular probes, long-lived lysosomes rather than newly acidified organelles are labeled. This is because once protonated within the acidic cell compartment, the net positively charged probes cannot easily cross the organelle′s membrane back into the cytosol. Thus, the concentration of probes outside of the lysosomes is typically one to two orders of magnitude lower than that inside the lysosomes, offering less chance for the staining of newly acidified organelles *in situ* during the experiment. Key to monitor successfully the newly acidified organelles thus is to ″smuggle″ molecular probes in an invisible, non-fluorescent form as ″sleeping agents″ into the cell and have them rather evenly distributed in the cytosol and organelles so that they can ″wake up″, or in other words transform into another visible, fluorescent form when an organelle acidifies. Ideally, for facile trafficking across membranes and within the cytosol both forms of the probe are charge-neutral or carry highly delocalized charges^36^, avoiding site-selective accumulation. Such a broad-band probe would naturally be rather non-specific with respect to different acidified organelles yet would be uniquely complimentary when used for instance in combination with a lysosome-specific stain.

Herein, we report a novel probe based on a pH-dependent reaction that takes place directly at the indacene core of a BODIPY dye. At neutral pH, the dye is in its colorless and non-fluorescent leuco-form. This saturates the *meso*-carbon and interrupts the BODIPY′s π-system. Upon acidification, at pH levels that are also characteristic of newly acidified organelles, it is transformed into the typical, all-π-conjugated BODIPY form that shows bright red color and fluorescence.

## Results

### Molecular design

**Fig. 1:**
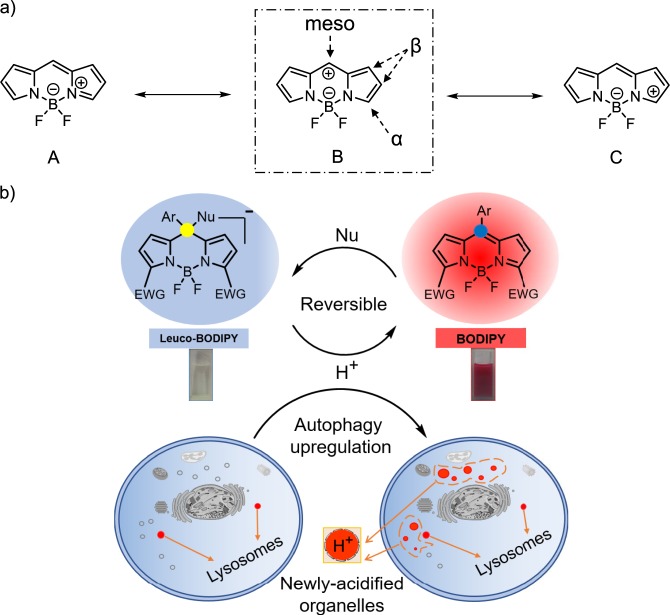
Schematic illustration of lighting up lysosomes and newly acidified organelles. **a** Potential mesomeric resonance structures of indacene skeleton. **b** Reversible transformation between BODIPY form in CH_2_Cl_2_ and leuco-BODIPY form in MeOH, and aspired signalling response during autophagy.

We based our molecular design on the three main mesomeric resonance structures of the indacene core, as shown in Fig. 1a. The formally positive charge is partly localized on the bridge head or *meso*-carbon and the degree of stabilization of the single structures thus depends on the pattern and electronic properties of substituents at the indacene core^37^. We thus prepared BODIPY **1** substituted with strongly electron-withdrawing groups (EWG) at the α-positions (Fig. 1b, Methods Section). The strongly electron-withdrawing effect of the substituents reinforces the electron deficiency at the respective ring positions, thus making especially the sp^2^ *meso*-carbon susceptible to attack by even only weakly nucleophilic agents. Such a reaction transforms this carbon into a sp^3^ *meso*-carbon, disrupting π-conjugation within the indacene system and leading to a dramatic fading of the color to colorless with a concomitant quenching of the red fluorescence (Fig. 1b). These pronounced spectroscopic differences between the π-conjugated fluorescent BODIPY and the saturated invisible leuco-BODIPY could dramatically reduce background fluorescence and improve signal-to-noise ratio. This strategy was previously reported by Urano′s group, who tuned the reactivity of an electrophile-containing rhodamine skeleton for the development of fast glutathione sensing probes^13^. In contrast to rhodamines, BODIPYs have long been recognized as robust fluorophores since post modification reactions often take place at the periphery rather than at the core^16–19^. The first example presumably showing a reaction at the BODIPY core was reported by Unaleroglu^20^, where an α-bis(methoxycarbonyl)ethenyl BODIPY underwent reversible protonation/deprotonation induced color and fluorescence changes, which was however mechanistically not explained or assigned to a reaction at the core. From the works of Urano and Unaleroglu, one can appreciate that EWG substitution at the α-positions of BODIPY can play a crucial role when aiming at a reaction at the BODIPY core. In our case, due to the lack of substituents at the 1,7-positions and the strong electron-withdrawing effect of the cyano groups at the 3,5-positions, the *meso*-carbon is prone to be attacked by weakly nucleophilic reagents such as methanol under neutral conditions.

### Photophysical properties

**Fig. 2:**
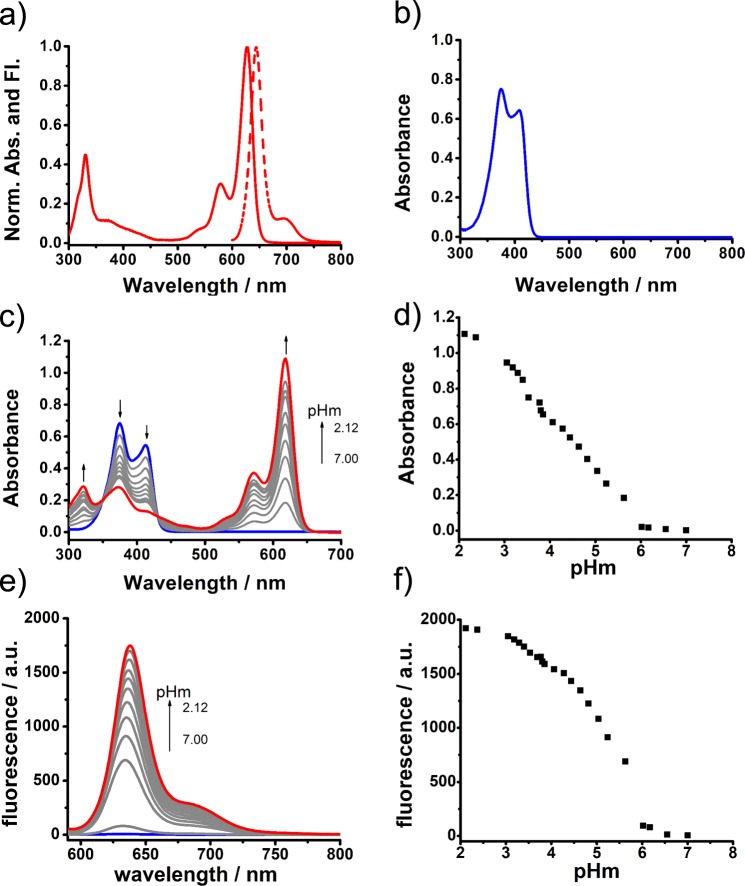
Absorption and fluorescence spectra of **1**. **a** In CH_2_Cl_2_. **b** In CH_3_OH. **c** Absorption spectral changes versus pHm in CH_3_OH. **d** The absorption changes at 618 nm during the pHm titration. **e** Fluorescence spectral changes versus pHm in CH_3_OH. **f** Fluorescence changes at 635 nm during the pHm titration. The concentration of **1** was 10^−5^ M for absorption and 10^-6^ M for fluorescence measurements. Definition of pHm: pHm, the apparent pH in methanol, is determined in analogy to pHe, a measure of the acid strength of ethanol and high ethanol content fuels as measured directly with a common pH electrode in high-content alcoholic solution, see ASTM D6423–08, ASTM International, West Conshohocken, PA (U.S.).

We investigated the spectroscopic properties of BODIPY **1** in solvents of different polarity (Supplementary Table 1). Figure 2 contains the absorption and fluorescence spectra of **1** in CH_2_Cl_2_ and CH_3_OH. The longest-wavelength absorption band in CH_2_Cl_2_ can be ascribed to the S_0_-S_1_ transition, with the typical 0–1 vibrational band as a shoulder at shorter wavelengths^38,39^. The introduction of the electron-withdrawing cyanoacrylate ester groups through exocyclic double bonds at the α-positions extends π-system delocalization and causes a red shift from 500 nm to 627 nm compared to the classical BODIPY dyes, very similar to distyryl extension (Fig. 2a)^40^. When the solvent is changed to methanol, the main BODIPY absorption band disappears completely, with two new bands appearing at 374 nm and 413 nm (Fig. 2b). This results in a visible color change from blue to virtually colorless and a quenching of the red fluorescence. Adding trifluoroacetic acid (TFA) to the methanol solution of **1** leads to the recovery of absorption and fluorescence bands as observed in CH_2_Cl_2_, e.g., from pale yellow to dark red upon going from pHm 7.0 to 2.1 (Fig. 2c, f; for a definition of pHm, see caption of Fig. 2). Closer inspection of the fluorescence changes as a function of proton concentration shows that the onset of fluorescence happens at ca. pHm 6, increasing ca. 20-fold until mid-point and finally resulting in a several hundredfold signal enhancement in the red spectral region (Fig. 2e, f). We further subjected **1** to spectroscopic titrations in water/ethanol 1:1 (v/v) mixtures, which is the best compromise for guaranteeing solubility of the dyes and comparability with the conventional pH scale effective in biological environments^41^. **1** showed the desired behavior also in mixed aqueous solution with a pK_a_ of 3.69 ( ± 0.05, corrected pK_a_, see ref. ^42^), and an apparent fluorescence quantum yield Φ_F_ of 0.21 at pH 4.5. In neat ethanol, however, **1** did not fade (Supplementary Fig. 1, 2), suggesting that water itself can act in the same way as CH_3_OH. To test the reversibility, we monitored changes in absorbance (at 380 nm and 620 nm) upon adjusting the pH alternatingly to be 4.0 and 7.4 in water/ethanol 1:1 (v/v). The efficiency of recovery lies at 100% (within uncertainty) after 10 cycles (Supplementary Fig. 3).

### Mass spectrometric and nuclear magnetic resonance studies

To obtain further insight into the molecular mechanisms at play, we interrogated the system by mass spectrometry (MS), ^1^H and ^13^C NMR. In the MS spectra, an anion signal at m/z 545.1816 was found and ascribed to [**1**-OCH_3_]^–^ (Supplementary Figs. 4, 5), indicating the formation of leuco-BODIPY.

**Fig. 3:**
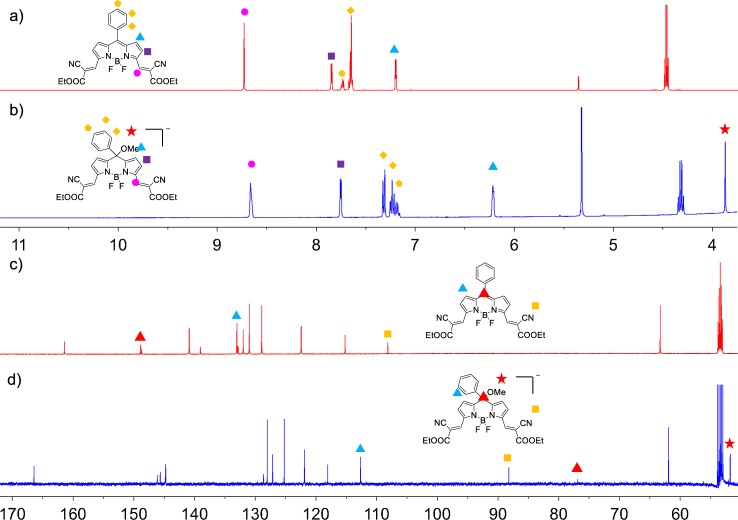
^1^H NMR spectra (600 MHz) of **1.** **a** In CD_2_Cl_2_, 298 K; **b** in CD_2_Cl_2_ containing 80 μL methanol (K_2_CO_3_ was added to remove any residue acid in the solvent), 298 K; ^13^C NMR spectra (150 MHz) of **1**: **c** in CD_2_Cl_2_, 298 K; **d** in CD_2_Cl_2_ containing 80 μL methanol (K_2_CO_3_ was added to remove any residue acid in the solvent), 298 K.

We measured ^1^H-NMR and ^13^C-NMR spectra in CD_2_Cl_2_ and CD_3_OD, using a 600 MHz NMR spectrometer. Due to the low solubility of **1** in deuterated methanol, a mixture of CD_2_Cl_2_/CD_3_OD (1:1, v/v) was used and a small portion of solid K_2_CO_3_ was added to remove any residual acid present in the solvent. We then measured two-dimensional NMR spectra to assign the signals (Supplementary Fig. 6–13). As shown in Fig. 3a, the proton signals of **1** in CD_2_Cl_2_ could be assigned with a COSY ^1^H-^13^C NMR spectrum. In CD_2_Cl_2_/CD_3_OD, the β-pyrrole proton closest to the *meso*-carbon shifted significantly up-field from 7.2 to 6.1 ppm (Fig. 3a, b), suggesting that the π-system of the indacene core is less extended after the formation of a tetrahedral geometry at the sp^3^ *meso*-carbon of the leuco-BODIPY. We also observed a similar up-field shift of the protons from the *meso*-phenyl ring (Fig. 3a, b). Clear evidence for the formation of the leuco-BODIPY form was found in the ^1^H- and ^13^C-NMR spectrum upon addition of 80 μL of MeOH (Fig. 3c, d), the structure of the BODIPY form completely converts into the leuco-BODIPY form with a new –OCH_3_ peak appearing at 3.8 ppm (see the whole spectra in Supplementary Fig. 14, 15). In addition, in the ^13^C NMR spectrum of BODIPY **1** in CD_2_Cl_2_ containing 80 μL MeOH, a new peak appeared at 72.38 ppm, assigned to the sp^3^ *meso*-carbon while the peak of the sp^2^ *meso*-carbon at 148.75 ppm in CD_2_Cl_2_ concomitantly disappeared. Also, a new peak at 51.77 ppm can be assign to the −OCH_3_ (Supplementary Table 2, Supplementary Fig. 16, 17). The *meso*-carbon is shifted for ca. 76 ppm up-field and the corresponding β-pyrrole carbons still for ca. 20 ppm; the chemical shifts of the other carbons are less than 7 ppm (Supplementary Table 2, Supplementary Figs. 16, 17). In order to verify the reversibility of the core-reaction at the molecular level, we added 10 μL trifluoroacetic acid (TFA) to the NMR tube, after which the proton peaks completely converted back to the BODIPY form and the –OCH_3_ peak at 3.8 ppm in ^1^H NMR as well as that at 51.77 ppm in ^13^C NMR disappeared (Supplementary Figs. 18, 19).

### Redox properties and theoretical calculations

Yang has evaluated the electronic interactions between EWGs and the BODIPY core by both electrochemical analysis and theoretical calculations^10^. In order to understand why the nucleophilic reaction occurs at the *meso*-position of **1**, theoretical calculations were performed on **1** and other BODIPYs with or without EWGs reported in the literature. In addition, we investigated the electrochemical properties of BODIPY **1** by cyclic voltammetry for reversible charge transfer and measured oxidation potentials by differential pulse voltammetry (DPV) for irreversible charge transfer. **1** showed two reversible reduction waves at half-wave potentials of –0.50 V (vs. ferrocenium/ferrocene) for the first reduction potentials and –1.10 V for the second reduction potentials (Supplementary Table 3, Supplementary Fig. 20), while an irreversible oxidation was observed at potentials of + 1.19 V (Supplementary Table 4, Supplementary Fig. 21).

The geometries for calculation were adopted from the crystal structure of **1** and optimized by the DFT method at the B3LYP/6–31 G(d) level of theory^43^. Reference compounds **2**^20^, **3**^10^ and **4**^10^ were also calculated to understand the contribution of the EWGs to making the *meso*-carbon position most prone to reaction. The optimized geometries with illustration of frontier orbitals of each compound are shown in Supplementary Fig. 22. The redox potentials and calculated molecular frontier orbitals are summarized in Supplementary Table 4. The LUMO levels increase on the order of **1** **→** **2** **→** **3** **→** **4**, reflecting the order of electron deficiency at the BODIPY core and reactivity toward nucleophiles. The calculated LUMO orbital distribution coefficients are included in Table 1.

**Table 1:** Selected molecular orbital coefficients (>0.1) of LUMO orbital of **1**.

Atoms^a^	Orbital coefficients absolute values		Orbital percentage (%)	Molecular structure
5 C (meso-carbon)	0.30501	0.25463	22.7	
7 C	0.15769	0.14575	6.6	
9 C	0.19469	0.16153	9.2	
10 C	0.10016	0.1033	3.0	
11 C	0.16900	0.16262	7.9	
26 N	0.15462	0.15009	6.7	

^a^The atom numbering is consistent with the X-ray numbering

### Cell imaging studies

**Fig. 4:**
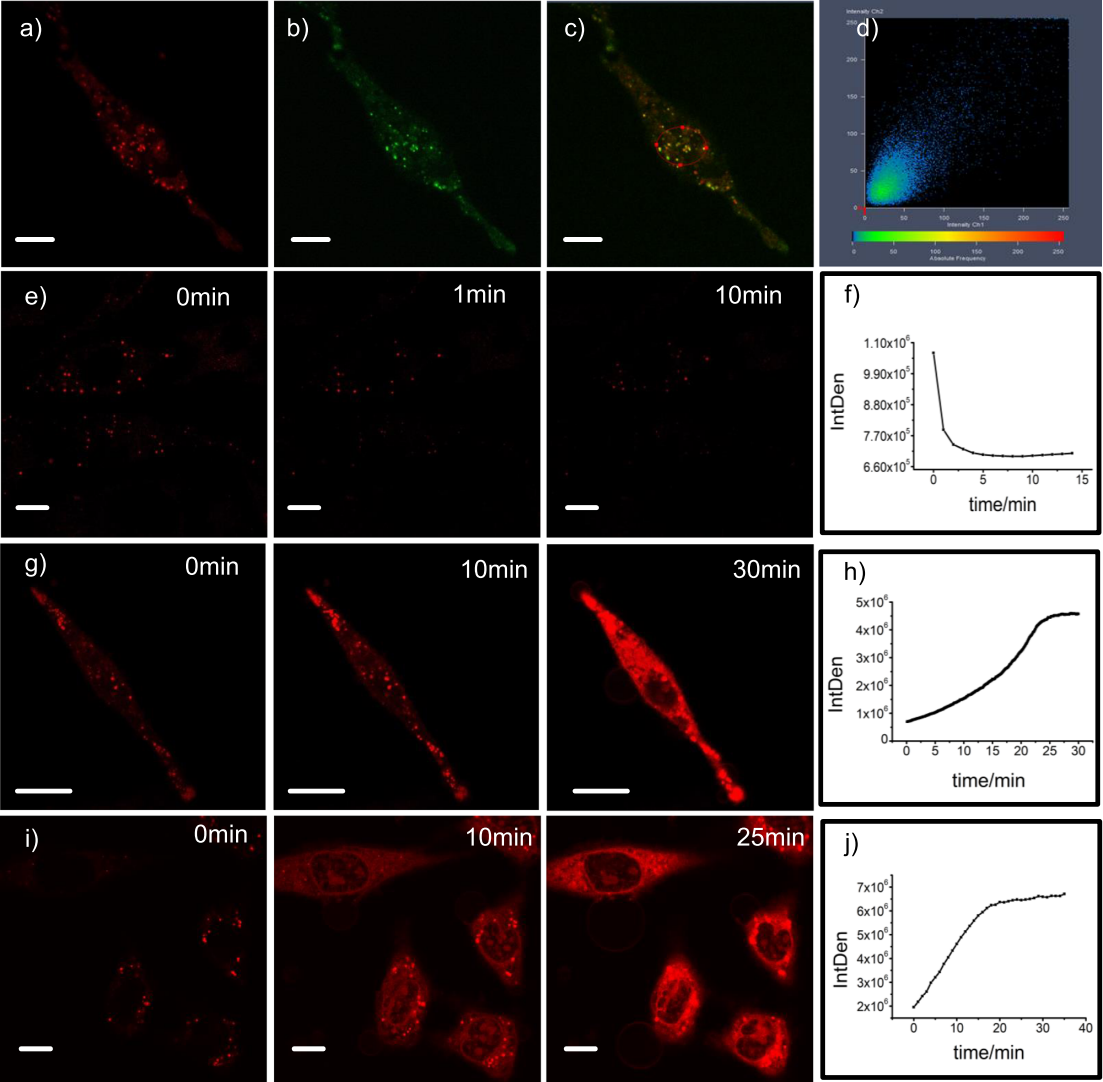
Real-time fluorescence imaging of lysosomes and cytosolic acidosis simultaneously. For **a**–**d**, Hela cells were co-cultured with **1** (10 µM) and LysoTracker Green (0.3 µM) for 30 min, then imaged under a microscope. **a** Cells were excited with a 543 nm laser, and the emission was collected between 580 and 730 nm. **b** Cells were excited with a 488 nm laser. **c** A merged image combining **a** and **b**. **d** Pearson intensity scatter plot of ROI. For panels **e**–**j**, Hela cells were loaded with **1** for 30 min and were submitted to confocal microscope observation followed by **e**, **f** addition of 10 μM chloroquine (images at different time intervals shown; plot of fluorescence intensity over time in **f**. **g** Addition of 10 μM dexamethasone (images at different time intervals shown); plot of fluorescence intensity over time in **h**. **i** *In-situ* incubation in a citric acid/phosphate buffer that was adjusted to pH ~4.5 and supplemented with nigericin and KCl (140 mM) (images at different time intervals shown); plot of fluorescence intensity over time in **j**. Scale bar 10 μm.

Having set the stage for reversible pH-driven interconversion of leuco-BODIPY and all-π-conjugated BODIPY forms mechanistically, we embarked on confocal fluorescence imaging experiments using **1** in living HeLa cells. Intense fluorescence was detected in lysosomes while negligible fluorescence was observed in other regions of the cell. The lysosomes in the image appear to be granular (spherical), which is consistent with the morphological features of lysosomes (Fig. 4a). The results were further confirmed by complete intracellular co-localization with the lysosome-specific counter stain LysoTracker Green (DND-26). **1** co-localized well with LysoTracker Green with a Pearson′s sample overlap coefficient of 0.90 (Supplementary Fig. 23), suggesting that **1** selectively images lysosomes in living cells (Fig. 4a–d).

Two drugs, chloroquine and dexamethasone, well-established in pH-related cell response studies^15,44^, were then used to alter lysosomal activity and cytosolic acidification. While the fluorescence intensity in the lysosomes was significantly reduced after addition of chloroquine and drug-induced increase in lysosomal pH (Fig. 4e, f), the addition of 10 μM dexamethasone led to a distinct increase of the fluorescence intensity of the cytosol, related to its acidification (Fig. 4g, h, supplementary movie 1).

To trace the conversion of the non-fluorescent leuco-BODIPY into the strongly fluorescent BODIPY form upon acidification of the cytoplasm we carried out a nigericin experiment. HeLa cells were loaded with **1** for 30 min and observed under the confocal microscope before incubating them in situ in a citric acid/phosphate buffer that was adjusted to pH ~4.5 and supplemented with nigericin and KCl (140 mM). This is an established method to equilibrate intracellular pH to an extracellular buffer^33^. As expected, we observed an obvious increase of red fluorescence in the cytosol in real time when the intracellular pH was reduced to pH ~4.5 (Fig. 4i, j).

To further assess the broad-band nature of the title dye, the dexamethasone experiments were additionally carried out in the presence of both LysoTracker Green and **1**. The set of images shown in Supplementary Fig. 24 reveals that whereas LysoTracker Green only locates within the lysosomes, dye **1** is widely distributed throughout the cells.

**Fig. 5:**
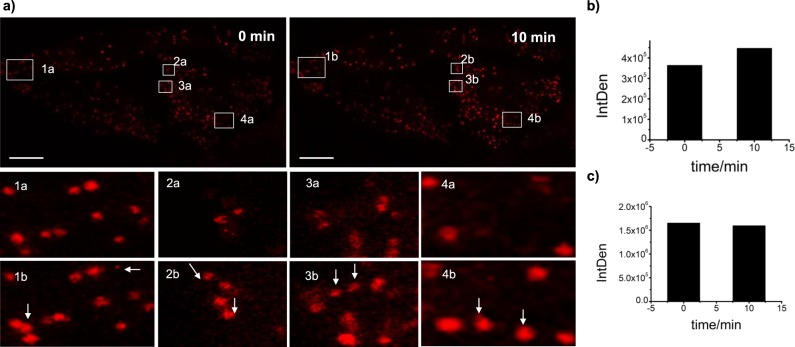
Real-time fluorescence imaging of newly acidified organelles during autophagy. **a** Hela cells were loaded with **1** (10 μM) for 30 min and were submitted to confocal microscope observation followed by addition of rapamycin (10 μM) under microscope. For each field, a series of 10–20 serial focal plane images was recorded, serial focal planes were 0.8 μm apart and were illuminated. Fluorescence images after selected time intervals, scale bar 10 μm. **b** Histogram of fluorescence intensity vs time upon addition of rapamycin. **c** Histogram of fluorescence intensity vs time without addition of rapamycin, scale bar 10 μm.

In the final step toward tracing autophagy, we carried out experiments involving rapamycin. Rapamycin promotes autophagy by inhibiting the mTORC1 complex^45^, which in turn is known to suppress the activity of the ULK1/2 complex that generally initiates autophagy^21–24^. As expected, upon addition of 10 μM rapamycin, new red fluorescent spots were observed in real time (Fig. 5a, supplementary movie 2). The total fluorescence intensity increased by 10% (Fig. 5b) while the background remained at zero (Supplementary Fig. 25), indicating that acidified organelles were formed but the cytosol was not acidified. As a control, we observed no increase in fluorescence intensity without the addition of rapamycin (Fig. 5c, Supplementary Fig. 26). As a second control, we performed the same experiment with LysoTracker Green. However, we did not observe any newly formed fluorescent spheres or increasing fluorescence intensity (Supplementary Fig. 27).

## Discussion

The dramatic changes in the absorption and fluorescence spectra of **1** observed by us are qualitatively similar to those reported previously on other reactive probes^10,13,20^. However, in contrast to Yang and co-workers, who systematically elaborated the mechanism how *meso*-unsubstituted BODIPYs with EWGs at the α-positions can form dimers by radical reactions upon addition of base^10^, our MS studies did not reveal any peaks belonging to dimers. Most probably, in our case, the phenyl group at the *meso*-position exerts moderate steric hindrance, preventing dimerization but allowing only attack of dimensionally small nucleophiles. In combination with NMR studies that furnished clear evidence for the formation of the leuco-BODIPY and its reversible interconversion into the all-π-conjugated BODIPY, we tentatively ascribe the molecular signaling mechanism of **1** to the nucleophilic attack of the *meso*-carbon of the rather electron-poor indacene core of **1** by methanol to form an sp^3^ carbon. The sp^3^ carbon disrupts the π-conjugated indacene plane and thus entails dramatic spectroscopic changes.

The electrochemical and theoretical data obtained here support this mechanism. The energy gap between the first reduction and the first oxidation potentials of **1** (1.69 V) is smaller by ca. 0.8 V than those of classical BODIPYs (2.30 V to 2.41 V)^10,46,47^, in good agreement with the ca. 130 nm red-shifted absorption maxima. Moreover, when compared to model dyes **2**–**4**, **1** contains the most electron-deficient *meso*-carbon, which reacts with MeOH already without addition of base. **2** and **3** only react upon addition of base, while **4** does not show any distinct spectroscopic change in the presence of a nucleophilic reagent. The trends of calculated HOMO, LUMO and their energy gaps match well with those reported previously by Hu et al.^10^ and measured by cyclic voltammetry, and are in agreement with our experimental results. Evaluation of the LUMO orbital distribution coefficients supports these findings. The atom with the highest priority for accepting electrons shall have the highest contribution, which indeed is the *meso*-carbon of **1** ( > 20% LUMO orbital contribution, Table 1). The contribution of each atom on the pyrrole rings is 5–10%, and it is < 5% for all other atoms. Because nucleophiles most preferentially attack those atoms that contribute the most to the LUMO orbitals, the probability for a reaction at the BODIPY core is highest at the *meso*-carbon.

The unique features of **1** as a probe for imaging intracellular acidification by way of simultaneously signaling the lighting-up lysosomes and cytosolic acidosis are directly linked to its strong emission in the red/NIR region under acidic conditions while being colorless in neutral aqueous solution. As was shown by the experiments involving chloroquine and dexamethasone, in cytoplasm or a neutral to alkaline organelle, the colorless leuco-BODIPY is the prevalent form, while the all-π-conjugated BODIPY form emits strong red fluorescence in an acidic organelle or the acidified cytosol. These results are consistent with literature reports^15^. Our data further indicate that the probe is reversibly responding to acidity in cellular environment and that, upon application of dexamethasone to the cells, cytosol acidification results in apoptosis^44^ (Fig. 4g, h, supplementary movie 1).

Many works have dealt with the real-time monitoring of lysosomes and cytoplasmic acidification, yet none of them have been reported to monitor the two processes simultaneously. However, many of the most pertinent biological questions are related to the interaction of more than one chemical species or the interplay of different pathways^48^. Currently, most lysosome-targeting probes are anchoring probes that usually consist of a masked fluorophore and a lipophilic weakly basic moiety. Upon protonation, the probes are trapped and accumulated in acidic vesicles due to the reduced membrane permeability^29,49^. However, as we have impressively demonstrated with the aid of nigericin, **1** differs from these probes in that a large number of probe molecules are latent in the cytosol in the form of the non-fluorescent leuco-BODIPY. Upon nigericin-mediated K^+^/H^+^ exchange and gradual acidification of the cytosol, these probe molecules are rapidly converted into the BODIPY form, emitting their strong fluorescence directly in the cytosol. The first set of cell experiments reported in this work clearly revealed that probe **1** is distributed in its invisible leuco form under neutral conditions within the entire cytosol and in its bright red-fluorescent BODIPY form in acidic environments (lysosomes), co-localized with a lyso-tracking dye. Upon acidification, when the pH of the neutral compartments of the cell drops, the colorless and non-fluorescent leuco-form is immediately converted into the red-fluorescent BODIPY form, allowing for real-time bioimaging of acidified organelles in living-cells.

**Fig. 6:**
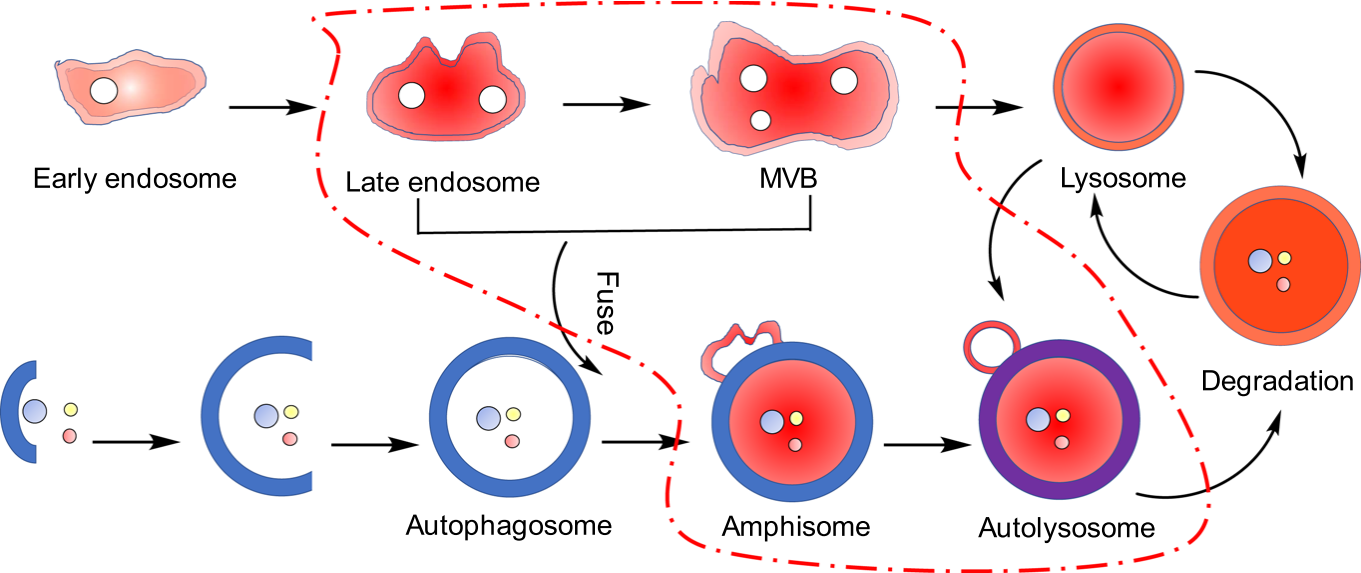
Schematic illustration of newly acidified organelles during autophagy. The steps encircled by the red dash-dotted line mark phases in which newly acidified organelles are formed.

The latter was demonstrated by the lighting-up of newly acidified organelles during autophagy. Autophagy is an intracellular degradative process by which cells control cellular homeostasis and survival^50^. It has been proposed that transient acidic organelles such as late endosomes are generated in the process of autophagy^21–24^. These objects have diameters of 250–1000 nm and a lumenal acidity of pH 4.9–6.0, which are similar to those of lysosomes^26^. As mentioned above, autophagy is initiated by the ULK1/2 complex, the activity of which is suppressed by the mTORC1 complex^21–24^, which itself is inhibited by rapamycin^45^. The experiments shown in Fig. 5, supplementary movie 2 and Supplementary Figs. 25–27 confirm this link between the observation of newly formed acidic organelles and autophagy, allowing for real time imaging. These newly formed acidic organelles might be late endosomes, multivesicular bodies or their fusion products, amphisomes, or subsequently matured products such as autolysosomes (Fig. 6). Discrimination between all these species solely on the basis of molecular probes is inherently difficult^21^. Although we could not specify the true nature or formation pathway of these objects in more detail, our approach accomplished to visualize these *in situ*-formed organelles, the leuco-BODIPY/BODIPY system providing a novel powerful tool for further studies of autophagy.

When attempting the real-time monitoring of newly acidified organelles, the probe not only showed good cell permeability and was readily taken up, but also negligible background fluorescence. This was achievable without any washing steps (Supplementary Figs. 23e, 23f, 28). Cytotoxicity tests showed that cell viability was always higher than 90% after incubation at different concentrations (0.1–100 μM) of **1**, indicating that the probe has negligible cytotoxicity to the cells (Supplementary Fig. 29).

In conclusion, we have reported to the best of our knowledge the first example of a reversible reaction-based signaling probe, involving the core of a BODIPY chromophore and taking advantage of a reversible nucleophilic reaction happening directly at the *meso*-carbon atom of indacene. This reaction allows to interconvert two persistently (meta)stable BODIPY forms solely by pH changes in the mildly acidic pH region. Whereas the leuco-BODIPY formed upon nucleophilic attack is a colorless and non-fluorescent species, the corresponding BODIPY is a brightly red emitting dye. Key to successful design was the introduction of two strongly electron-withdrawing groups at the α-positions of the BODIPY core. The π-disrupted leuco-BODIPY absorbs only in the UV-region and can be reversibly converted into the deeply colored and strongly red-emitting BODIPY at pH ~4. This proton-dependent equilibrium makes the dye suitable for use as a fluorescent turn-on probe for broad-band intracellular acidification as well as for monitoring acidic organelles. Unlike most reported amino-containing probes for acidic organelles, both forms of **1** are either net charge-neutral (only intrinsically zwitterionic at the B–N motif) or delocalized anionic and should thus show rather similar membrane permeability while being distributed in the entire cytosol, allowing for instant conversion wherever acidification occurs without being selectively accumulated in cell compartments. The excellent (meta)stability of the two forms of the dye render BODIPY **1** a potent tool for real-time fluorescence monitoring of lysosomes and cytosolic acidosis simultaneously, as well as the detection of newly acidified organelles in autophagic pathways. The excellent cell permeability and negligible background fluorescence outside of the target organelles allows investigators to dispense with additional washing steps, contributing to the benefits of **1**. Keeping in mind the versatility of BODIPY chemistry, we expect that our present research on a core-reactive BODIPY platform will inspire the development of more advanced functional probes.

## Methods

### General

The HeLa cell line was bought from Synthgene Co., Nanjing. All reagents were obtained from commercial suppliers and used without further purification unless otherwise indicated. All oxygen-, water- or light-sensitive reactions were carried out under nitrogen atmosphere in oven-dried glassware or covered by aluminum foil. Glassware was dried in an oven at 120 °C and cooled under a stream of inert gas before use. When dichloromethane or chloroform is specified, these were distilled over calcium hydride. Triethylamine was obtained by simple distillation. ^1^H and ^13^C NMR spectra were obtained in the indicated solvents, with Bruker DRX400, DRX500 or DRX600 spectrometers. ^13^C spectra were ^1^H decoupled. Chemical sifts are given in ppm with the residual solvent peaks used as the reference signals. Coupling constants are given in Hz. High resolution mass spectra were measured on Agilent 6540 Q-TOF LC/MS. Cyclic voltammetry experiments were carried out on BAS E2P electrochemical workstation. All the solvents employed for the spectroscopic measurements were of UV spectroscopic grade (Aldrich).

### General synthesis of 1

**Fig. 7:**
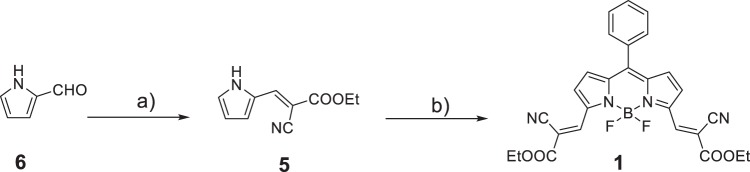
Synthetic procedures and molecular structures of BODIPY **1**. **a** Ethyl 2-cyanoacetate, hexamethylenetetramine; reflux in dry toluene for 2 h; **b** benzaldehyde, trifluoroacetic acid, 2,3-dichloro-5,6-dicyano-1,4-benzoquinone, triethylamine, BF_3_•Et_2_O.

BODIPY **1** substituted with strongly electron-withdrawing diethyl 2-cyanoacrylate groups at the α-positions was prepared via a conventional TFA-catalyzed condensation reaction of benzaldehyde with ethyl(*E*)-2-cyano-3-(1*H*-pyrrole-2-yl)acrylate **5**, followed by oxidation with 2,3-dichloro-5,6-dicyano-1,4-benzoquinone (DDQ) and cyclization with BF_3_·Et_2_O (Fig. 7). **5** was prepared by reaction of ethyl 2-cyanoacetate with 1*H*-pyrrole-2-carbaldehyde **6**. The structure of **1** was confirmed by ^1^H NMR, ^13^C NMR spectroscopy and HRMS.

### Synthetic details of 1 and 5

Benzaldehyde (2 mmol) and 2-(2-cyano-2-ethoxycarbonylvinyl)-1H-pyrrole (2 mmol) were dissolved in dry dichloromethane (50 mL) under nitrogen, covered by aluminum foil. 0.5 mL Trifluoroacetic acid (TFA) was added. Refluxing and stirring lasted for 4 days until the pyrrole was completely consumed, as indicated by TLC. Dichlorodicyanobenzoquinone (DDQ, 2 mmol) was added, and the mixture was stirred for 30 min at room temperature. Triethylamine (TEA, 3 mL) and boron trifluoride etherate (10 mL) were added. After 8 h of refluxing, the blue solution (with strong red fluorescence) was washed with water, saturated sodium bicarbonate solution, and then dried over anhydrous magnesium sulfate and concentrated under reduced pressure. Purification was undertaken by silica gel column chromatography using dichloromethane. The compound was recrystallized from dichloromethane/hexane.

Pyrrole **5** was prepared from the reaction of ethyl 2-cyanoacetate with 1*H*-pyrrole-2-carbaldehyde and hexamethylenetetramine under reflux in dry toluene for 2 h. It was recrystallized from dicholoromethane and hexane.

### Characterization of 1 and 5

BODIPY **1** was obtained as green metallic crystals (298.2 mg, 58% yield).^1^H NMR (600 MHz, CD_2_Cl_2_) δ = 8.74 (s, 2 H), 7.85 (d, *J* *=* 4.7, 2 H), 7.78–7.71 (m, 1 H), 7.70–7.62 (m, 4 H), 7.21 (d, *J* = 4.7, 2 H), 4.47 (q, *J* *=* 7.1, 4 H), 1.46 (t, *J* *=* 7.1, 6 H). ^13^C NMR (150 MHz, CD_2_Cl_2_) δ = 161.44, 148.90, 148.75, 140.88, 139.04, 133.02, 132.83, 131.98, 130.98, 128.93, 122.45, 115.22, 108.17, 63.32, 53.79, 53.61, 53.43, 53.25, 53.07, 13.91, −0.43. HRMS (ESI) m/z calcd for C_27_H_21_BF_2_N_4_O_4_Na 537.1516; found: m/z [M + Na]^+^ 537.1513.

**5** was obtained as yellow needles (2.55 g, yield 72.9%). ^1^H NMR (500 MHz, DMSO) δ = 9.95 (s, 1 H), 8.03 (s, 1 H), 7.26 (ddd, *J* *=* 3.2, 2.5, 0.8, 1 H), 6.97 (s, 1 H), 6.45 (dt, *J* *=* 3.9, 2.4, 1 H), 4.35 (q, *J* *=* 7.1, 2 H), 1.39 (t, *J* *=* 7.1, 3 H).

### Crystal structures of 1 and 5

Single crystals of **1** and **5** suitable for X-ray structural analysis were obtained by slow diffusion of hexane into a chloroform solution. Their crystal structures and molecular packings are shown in Supplementary Fig. 30, 31. The cell parameters and refinement details are summarized in Supplementary Table 5. The molecules are packed in a face-to-tail orientation along the crystallographic c-axis, the distance between two indacene ring planes being larger than 3.7 Å, thus no π-π stacking can be observed. Intermolecular hydrogen bonds can be found between carbonyl oxygen and pyrrole hydrogen. The central six-membered ring of **1** containing B–N1A–C6A–C5–C6–N1 is almost coplanar with the adjacent five-membered rings with the average deviation from the mean plane being 0.153 Å, indicating strong π-electron delocalization within the indacene plane. The bond length of C10–C11 is 1.345 Å, indicating a double bond character. Due to the absence of substituents on the β-pyrrole moieties, the dihedral angle between the *meso*-phenyl ring and the indacene plane is only 48.9°, significantly smaller than that for many classical tetra-alkyl-BODIPYs in which the *meso*-phenyl ring is almost perpendicularly oriented to the indacene plane with torsion angles > 80°^51^.

### Steady-state absorption and fluorescence spectroscopy

Steady-state absorption and fluorescence measurements were carried out on Specord 210 plus (Analytik Jena AG) and Bruins Instruments Omega10 spectrophotometers as well as Spectronics Instruments 8100 and Horiba Jobin-Yvon Fluoromax-4P fluorometers, employing UV-spectroscopic solvents. For all measurements, the temperature was kept constant at 298 ± 1 K and, except where noted, dilute solutions with an absorbance of less than 0.1 at the absorption maximum were used, especially for fluorescence measurements. The latter were performed with a 90° standard geometry. The fluorescence quantum yields (Φ_f_) of **1** was determined relative to oxazine 170 in ethanol (Φ_f_ = 0.58 ± 0.02)^52^. The uncertainties of measurement were determined to ± 5 % (for Φ_f_ > 0.2).

### Time-resolved fluorescence spectroscopy

Fluorescence lifetimes (τ_f_) were determined with a unique customized laser impulse fluorometer with picosecond time resolution described in ref. ^53^. The fluorescence was collected at right angles (monochromator with spectral bandwidths of 4 and 16 nm) and the decays were recorded with a modular single photon timing unit and a time division of 4.8 ps channel^–1^. Typical instrumental response functions with a full width at half maximum (*fwhm*) of ca. 25–30 ps were realized, resulting in an uncertainty of ±3 ps, respectively. Attenuation of the laser beam was accomplished with a double prism attenuator from LTB and typical excitation energies were in the nanowatt to microwatt range (average laser power). The fluorescence lifetime profiles were analyzed with a PC using the software package Global Unlimited V2.2 (Laboratory for Fluorescence Dynamics, University of Illinois). The goodness of the fit of the single decays as judged by reduced chi-squared (*χ*_R_^2^) and the autocorrelation function *C(j)* of the residuals was always below *χ*_R_^2^ < 1.2. For all the dyes, decays were recorded at three different emission wavelengths over the BODIPY-type emission spectrum and analyzed globally. Such a global analysis of decays recorded at different emission wavelengths implies that the decay times of the species are linked while the program varies the pre-exponential factors and lifetimes until the changes in the error surface (*χ*^*2*^ surface) are minimal, that is, convergence is reached. The fitting results are judged for every single decay (local *χ*_*R*_^*2*^) and for all the decays (global *χ*_*R*_^*2*^), respectively. The errors for all the global analytical results presented here were below a global *χ*_*R*_^*2*^ = 1.2.

### pH-dependent spectroscopic properties

For every step of the pH titration, small amounts (2–10 µL) of HClO_4_ solutions of appropriate concentration (1, 0.1, 10^–2^, and 10^–3^ M) in water/ethanol 1:1 (v/v) were added (microliter pipette, Eppendorf) directly into the measurement cell, filled up with a solution (2 mL) containing the dye (2 µM) in a mixture of water/ethanol 1:1 (v/v). The pH was monitored at 298 K using a digital pH meter (Metrohm 827 pH lab) equipped with a Biotrode (Metrohm). Calibration of the instrument was performed with standard aqueous solutions of pH 4, 7, and 9 from Metrohm. The measured pH value was corrected by taking into account differences in liquid junction potentials and proton activity coefficients between the solvent mixture of the sample and the aqueous calibration solution according to a procedure described in detail in ref. ^42^. p*K*_a_ data were determined from at least two replicate measurements.

### General cell culture and imaging

HeLa cells were grown in Dulbecco′s Modified Eagle′s Medium (DMEM) media supplemented with 10% fetal bovine serum (FBS) and 1% 100 U mL^–1^, 100 mg L^–1^ streptomycin in glass bottom dishes. Cells were cultured under a controlled atmosphere (37 °C, 5% CO_2_) for 24 h. The medium was removed and washed 3 times with Hank’s Balanced Salt Solution (HBSS), then **1** (10 µM, dissolved in HBSS with 1% DMSO for solubilization) was added and incubated for 30 min. Confocal images were acquired with a confocal laser scanning microscopy (Zeiss, LSM710). A 37 °C incubator was used during bioimaging. Two channels were used in co-localization experiments.

### Real-time detection of newly acidified organelles

In order to avoid observation errors caused by cell deformation or organelle movement, a series of 10–20 serial focal plane images was recorded for each field, serial focal planes were 0.8μm apart and were illuminated. The image shown in the article is a vertical projection of all planes.

### Software

Image J, Zeiss Zen 2008.

### Statistics and reproducibility

The number of cells available for analysis varied from experiments due to the variation on magnification, resolution and stimulation method. To ensure data quality and reproducibility, all the imaging experiments were repeated at least twice, and at least two biological replicates were analyzed for each imaging experiment. Cells were allocated randomly into experimental groups.

### Reporting summary

Further information on research design is available in the Nature Research Reporting Summary linked to this article.

## Supplementary information

Supplementary Information

Descriptions of additional supplementary files

Reporting Summary

Supplementary Movie 1

Supplementary Movie 2

## Data Availability

The data that support the findings of this study are available from the corresponding author upon reasonable request. Synthetic procedures are detailed in the Methods. Crystal data, spectral characterization and cell imaging data of **1** are provided in the Supplementary Information file. Supplementary Movie 1 shows the real-time imaging of Hela cells after incubation with **1** (10 µM) for 30 min upon addition of dexamethasone (10 µM). Supplementary Movie 2 shows real-time imaging of Hela cells after incubation with 1 (10 µM) for 30 min upon addition of rapamycin (10 µM). Crystallographic data for CCDC-1551499 for **1** and CCDC-843444 for **5** are provided in the Supplementary Information file. These data can be obtained free of charge at www.ccdc.cam.ac.uk/conts/retrieving.html [or from the Cambridge Crystallographic Data Centre, 12, Union Road, Cambridge CB2 1EZ, UK; fax: (internat.) + 44 1223/336 033; E-mail: deposit@ccdc.cam.ac.uk].
