# Supplementary material for: Real-time monitoring of newly acidified organelles during autophagy enabled by reaction-based BODIPY dyes
Source: Commun Biol. 2019 Nov 28;2:442. doi: 10.1038/s42003-019-0682-1 (PMC6883057; doi:10.1038/s42003-019-0682-1)
Supplement: Supplementary file 1 — Supplementary Information [file 42003_2019_682_MOESM1_ESM.pdf]

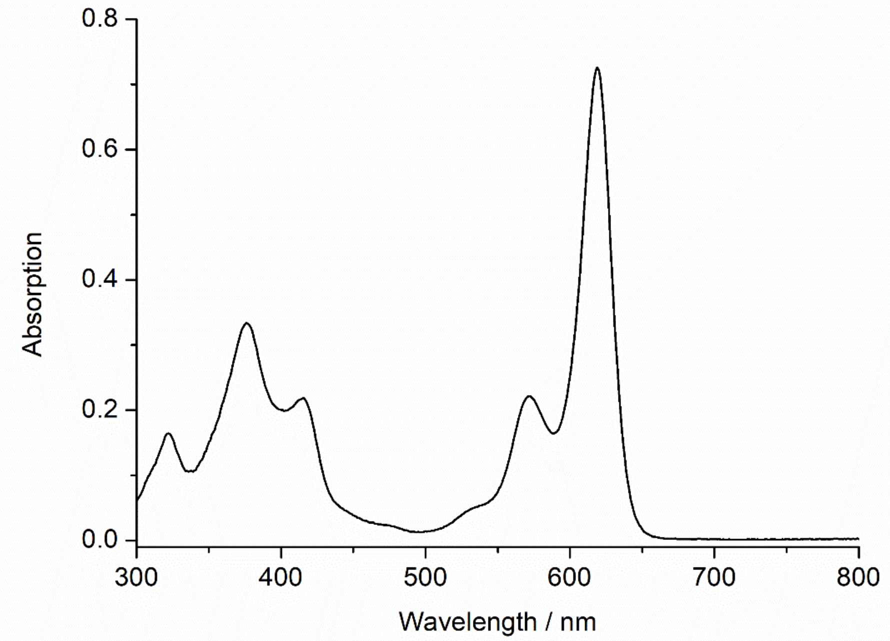

Supplementary Figure 1. Absorption spectrum of **1** in neat ethanol (100%). The concentration of **1** is 10 μM.

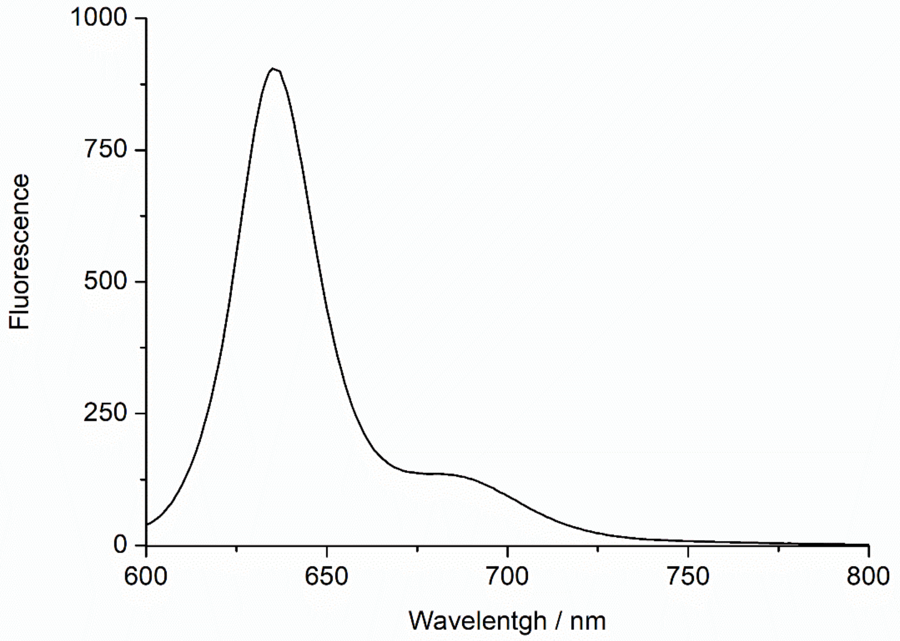

Supplementary Figure 2. Fluorescence spectrum of **1** in neat ethanol (100%). The concentration of **1** is 10 μM. The excitation wavelength is 543 nm.

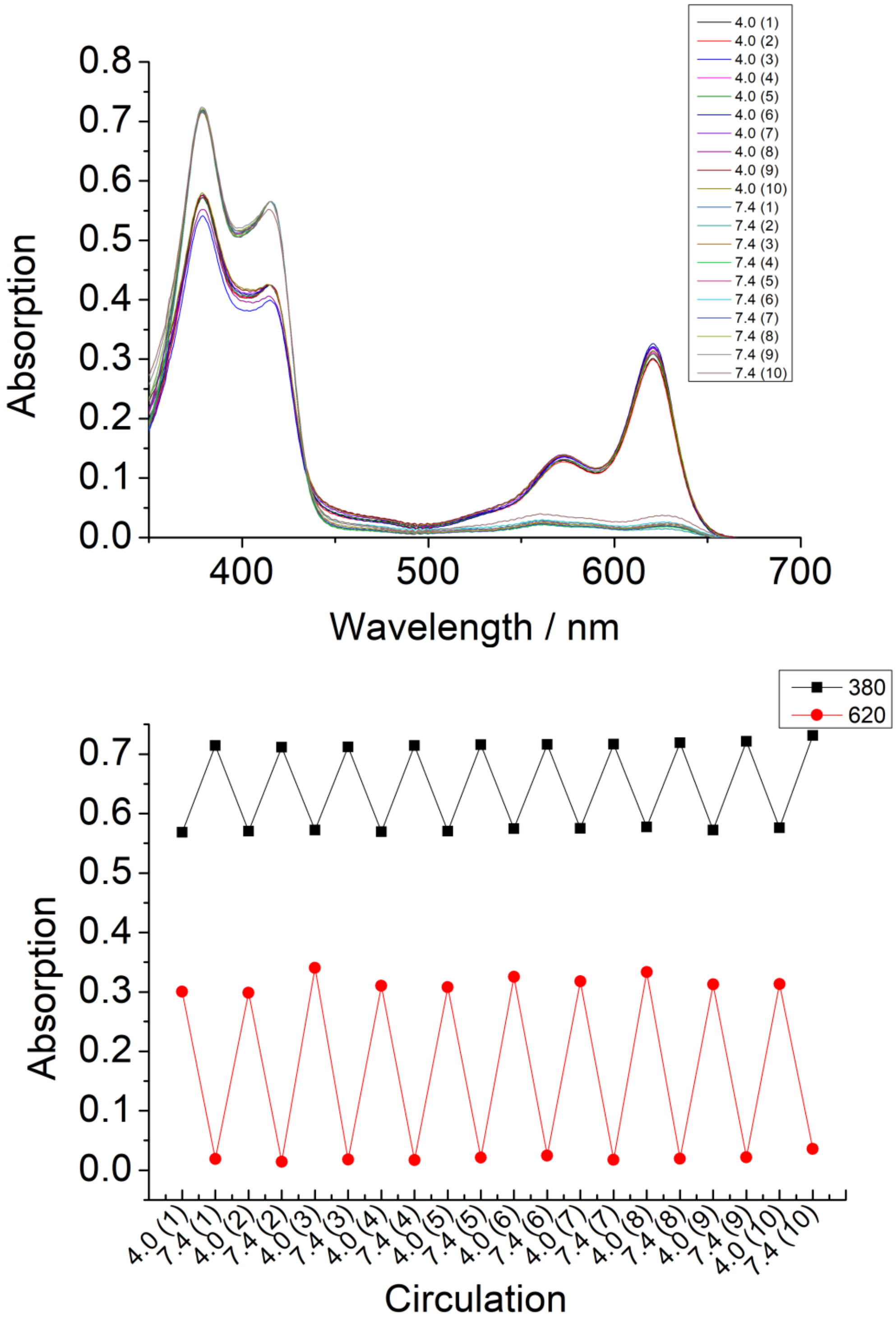

Supplementary Figure 3. Reversibility test of 1 by apsoprption spectra. (a) The absorption spectra of **1 (**10^{-5} M in ethanol/H_2O (*v*/*v* = 1:1)) recorded at pH = 4 and 7.4, respectively, for 10 cycles (The pH value was sequentially adjusted by addition of trifluoroacetic acid and 1,8-bis(dimethylamino)naphthalene and monitored with a pH meter). (b) The absorption maximum at 380 nm (black) corresponding to the Leuco-BODIPY and 620 nm (red) corresponding to the BODIPY form were selected for comparison.

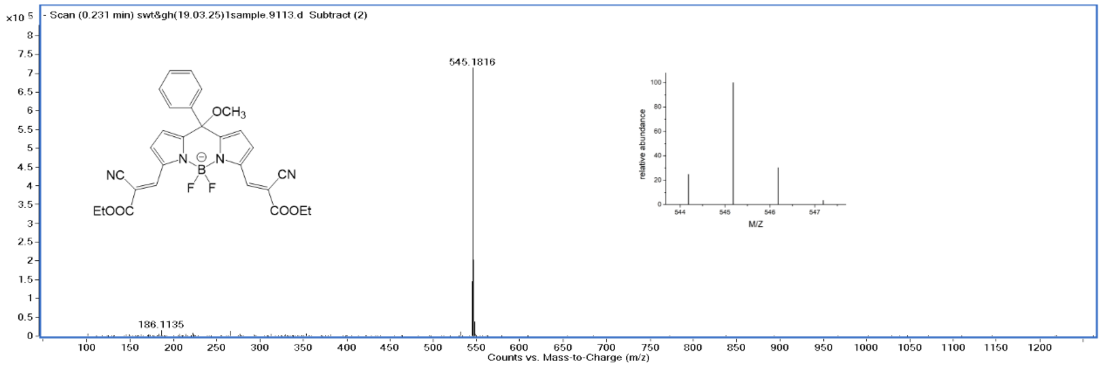

Supplementary Figure 4. HRMS spectrum of BODIPY **1** in CH_3OH (measured in anion mode. K_2CO_3 was added to remove any residual acid from the solvent).

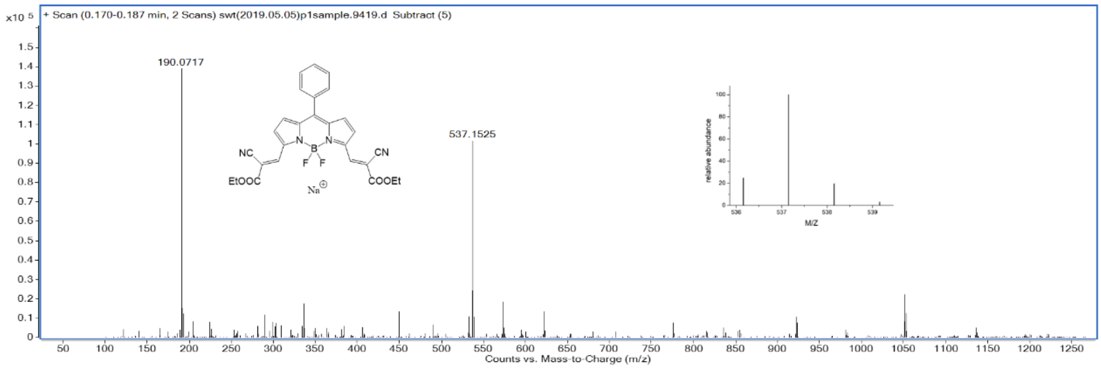

Supplementary Figure 5. HRMS spectrum of BODIPY **1** in CH_2Cl2.

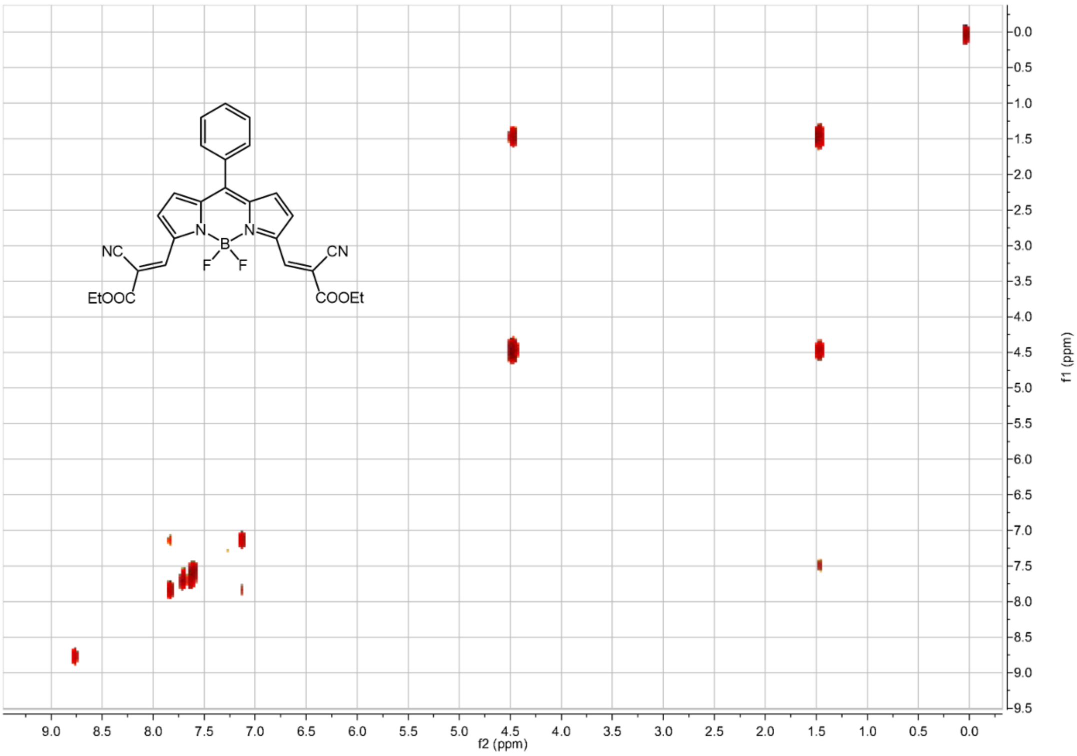

Supplementary Figure 6. H-H COSY spectrum of **1** in CD_2Cl_2 at room temperature.

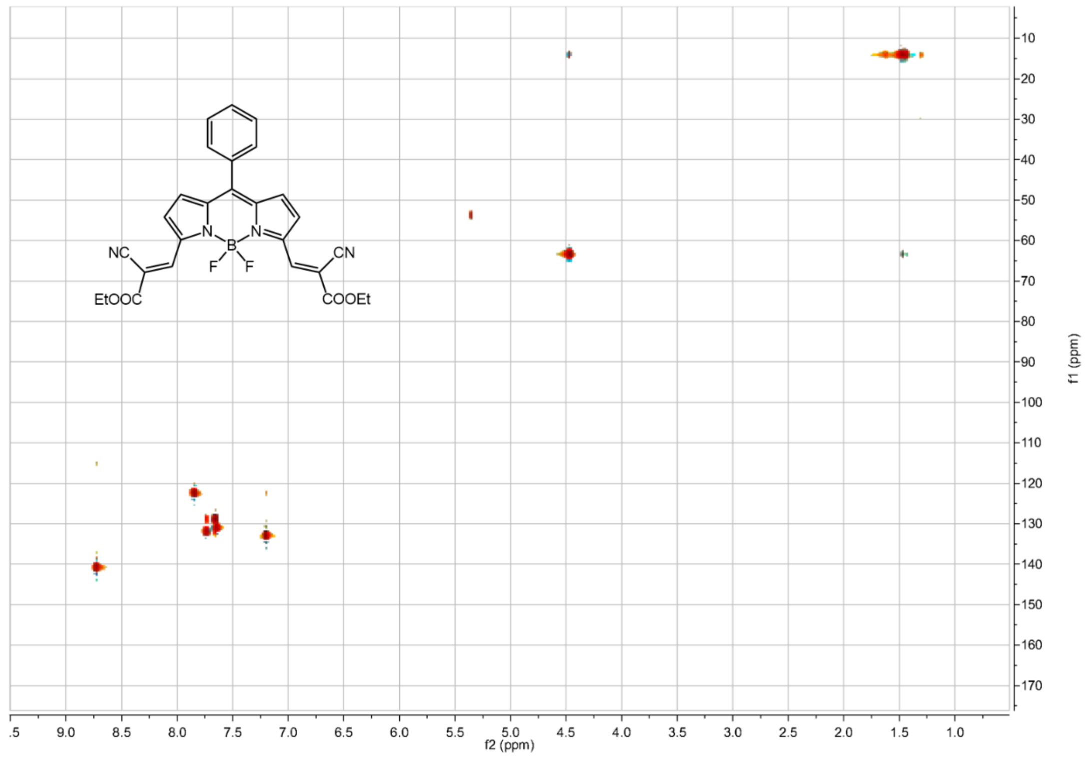

Supplementary Figure 7. C-H COSY spectrum of **1** in CD_2Cl_2 at room temperature.

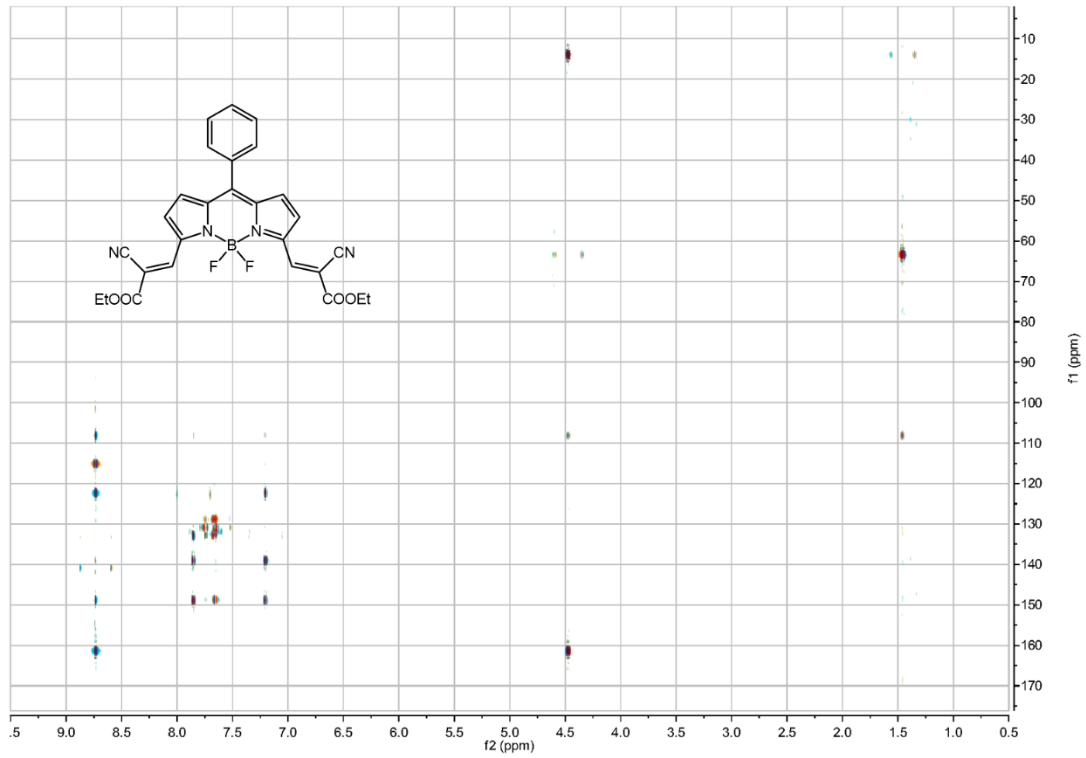

Supplementary Figure 8. HMBC spectrum of **1** in CD_2Cl_2 at room temperature.

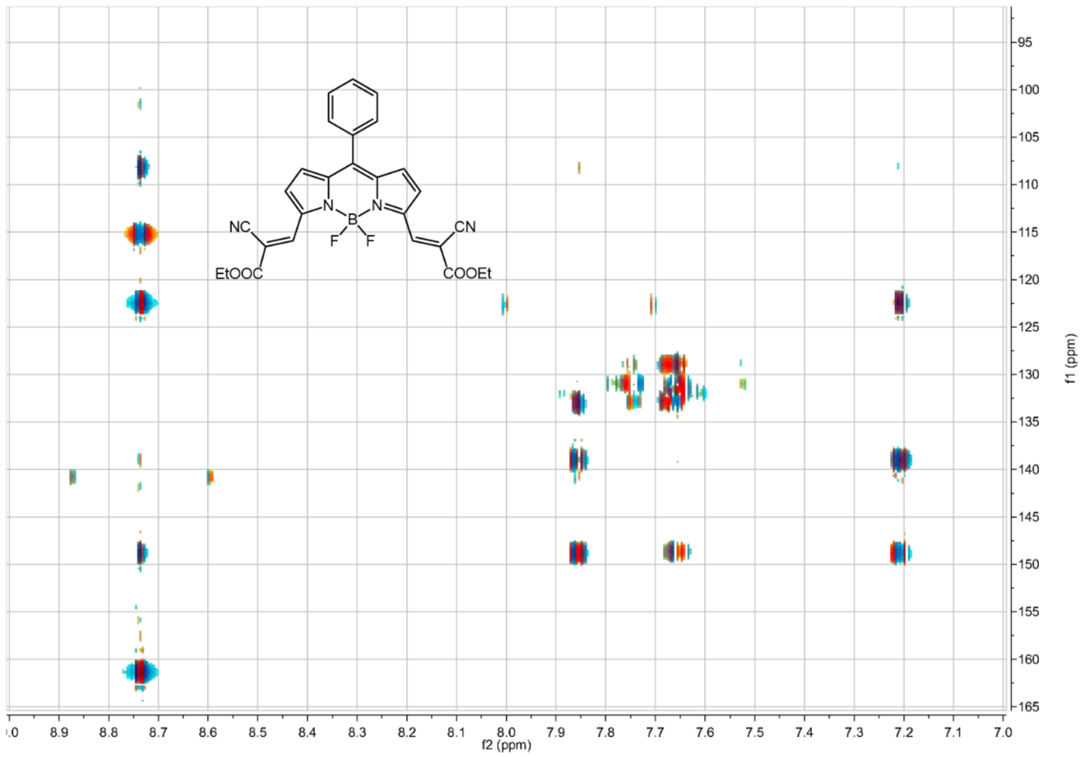

Supplementary Figure 9. HMBC spectrum (zoom) of **1** in CD_2Cl_2 at room temperature.

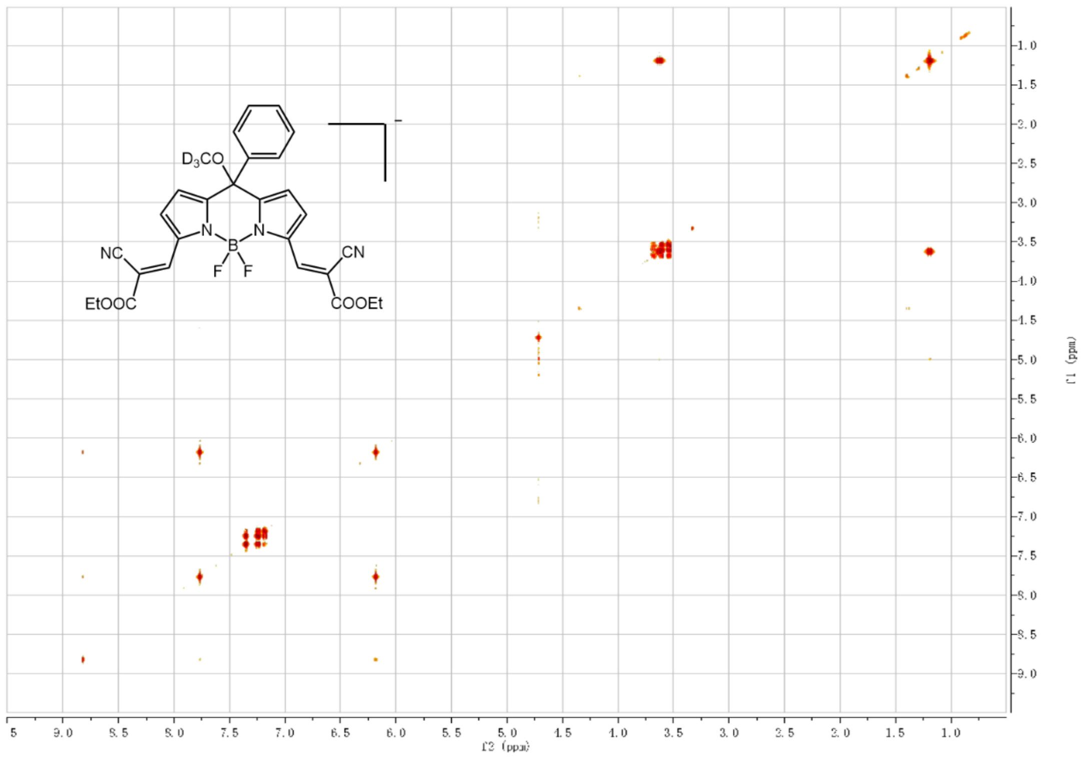

Supplementary Figure 10. H-H COSY spectrum of **1** in CD_3OD+CD_2Cl_2+K_2CO_3 at room temperature.

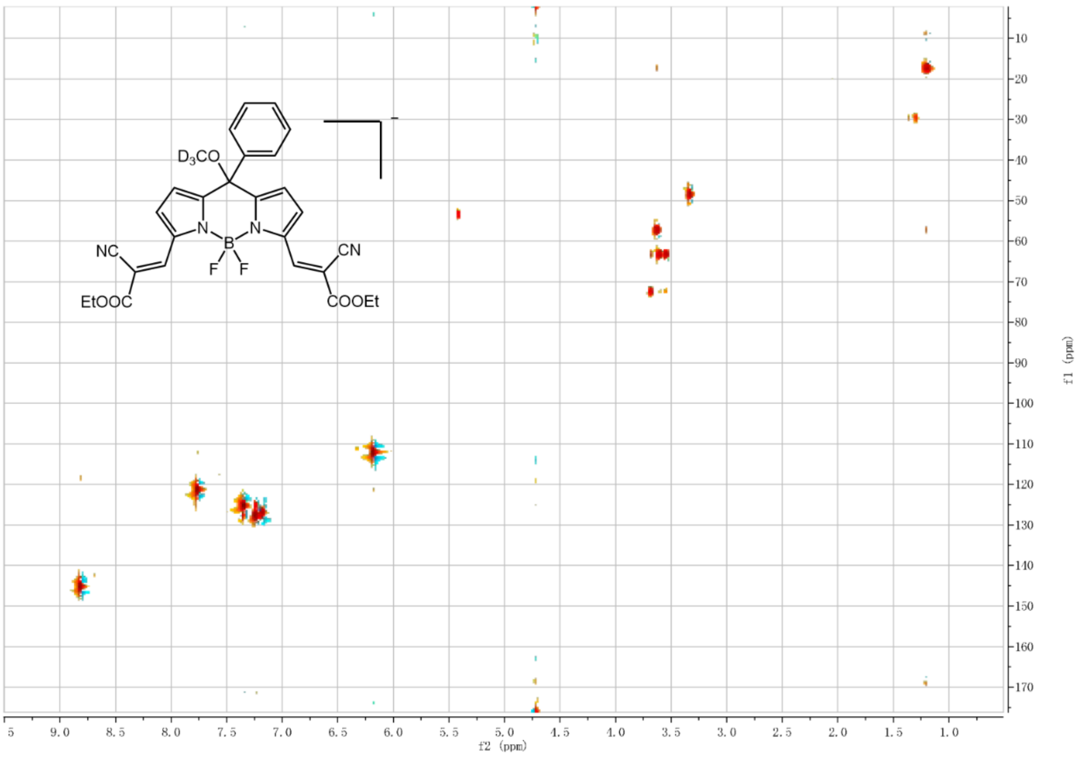

Supplementary Figure 11. C-H COSY spectrum of **1** in CD_3OD+CD_2Cl_2+K_2CO_3 at room temperature.

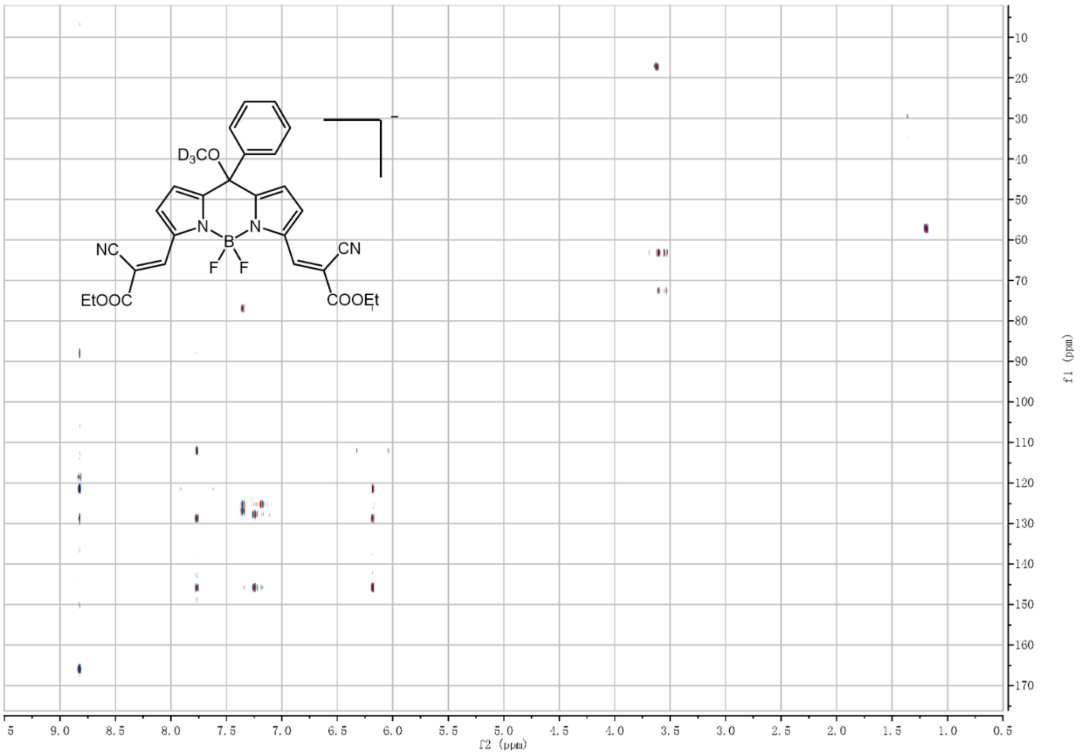

Supplementary Figure 12. HMBC (^{1}H detected heteronuclear multiple bond correlation) spectrum of **1** in CD_3OD+CD_2Cl_2+K_2CO_3 at room temperature.

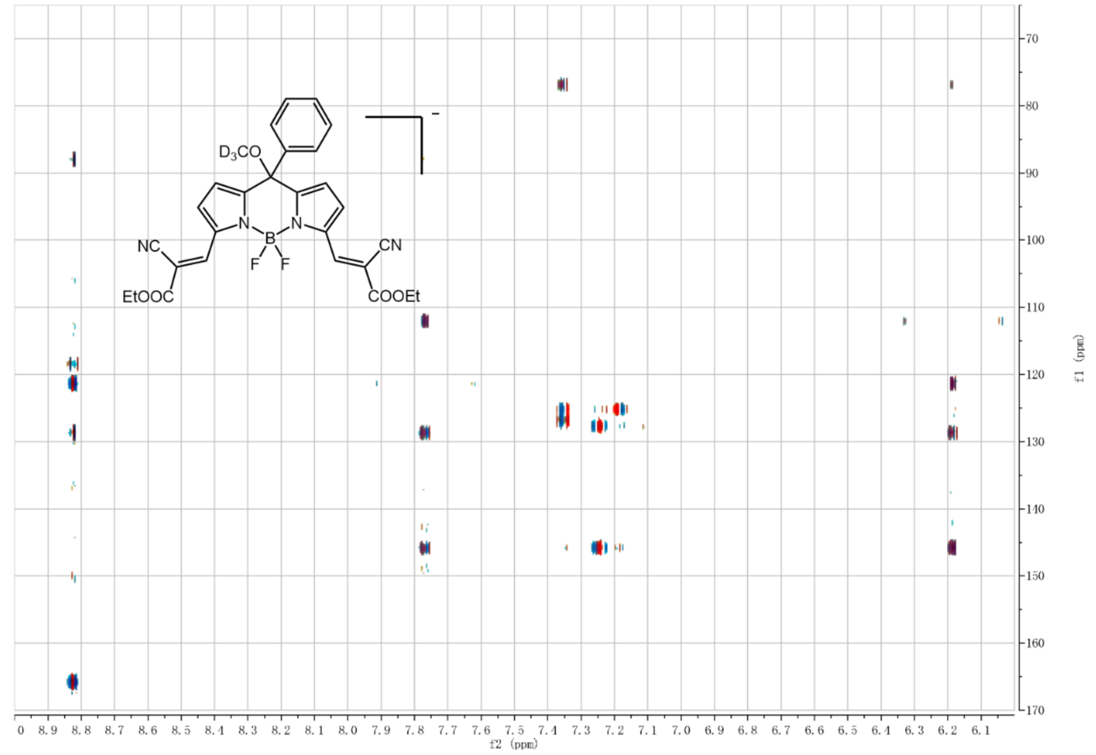

Supplementary Figure 13. HMBC spectrum (zoom) of **1** in CD_3OD+CD_2Cl_2+K_2CO_3 at room temperature.

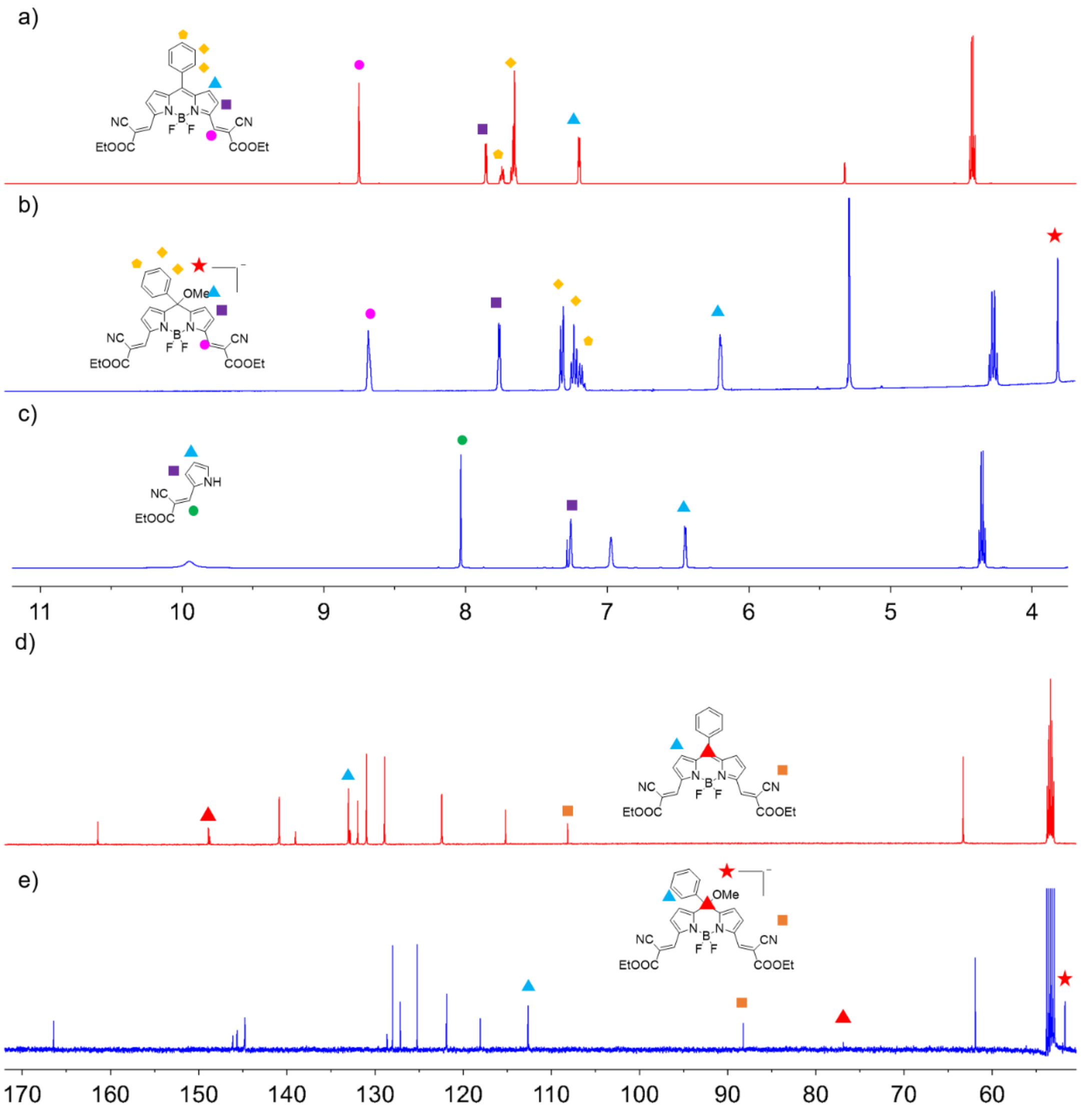

Supplementary Figure 14. ^{1}H and ^{13}C NMR spectra of **1** in CD_2Cl_2 and those upon addition of 80 μL CH_3OH in the presence of K_2CO_3 at room temperature. a) in CD_2Cl_2, 298 K; b) in CD_2Cl_2 upon addition of 80 μL CH_3OH in the presence of K_2CO_3, 298 K.; c) pyrrole **2** in $CDCl_3$, 298 K; ^{13}C NMR spectra (150 MHz) of **1**: d) in CD_2Cl_2, 298 K; e) in CD_2Cl_2 upon addition of 80 μL CH_3OH in the presence of K_2CO_3, 298 K.

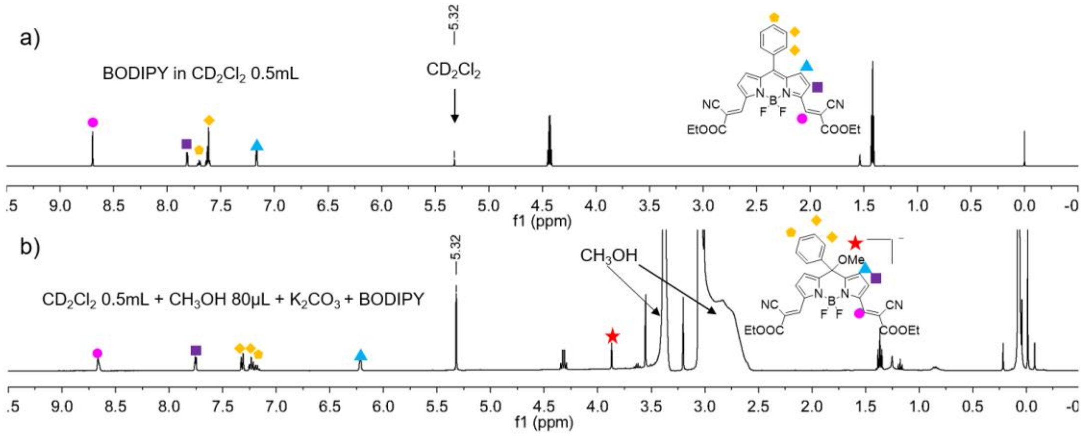

Supplementary Figure 15. Whole spectra of 1H NMR spectra of **1** in CD2Cl2 that upon addition of 80 μL CH3OH in the presence of K2CO3. a) in CD_2Cl_2; b) in CD_2Cl_2 upon addition of 80 μL CH_3OH in the presence of K_2CO_3, 298 K.

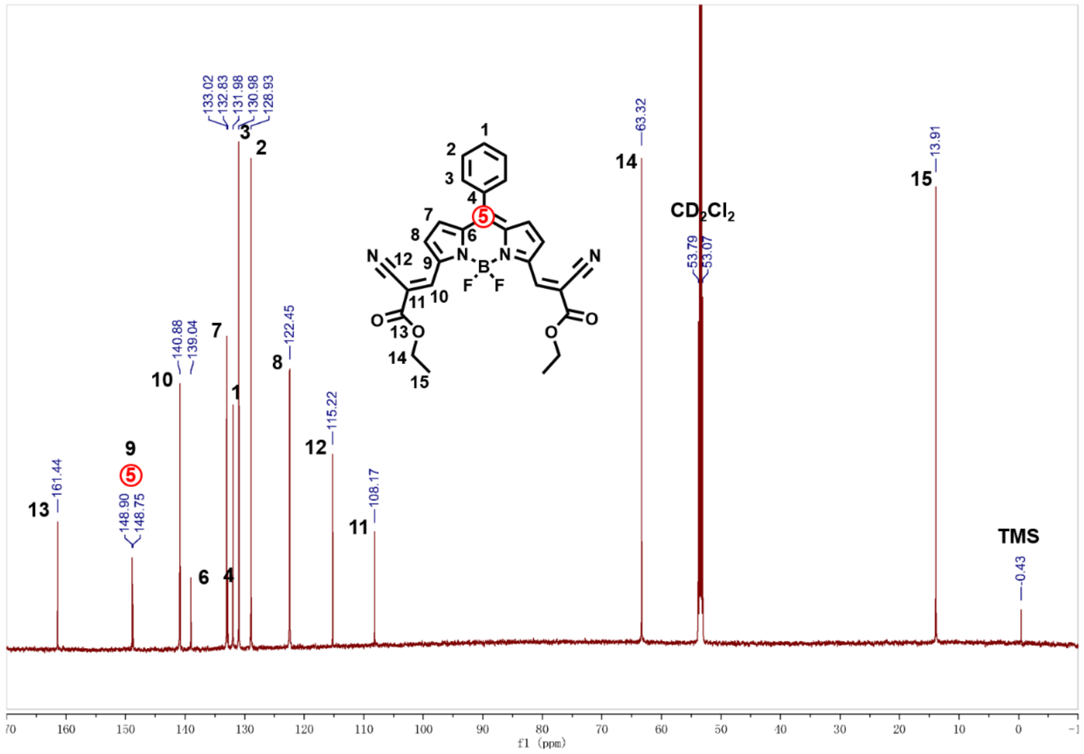

Supplementary Figure 16. ^{13}C NMR of **1** in CD_2Cl_2 at room temperature.

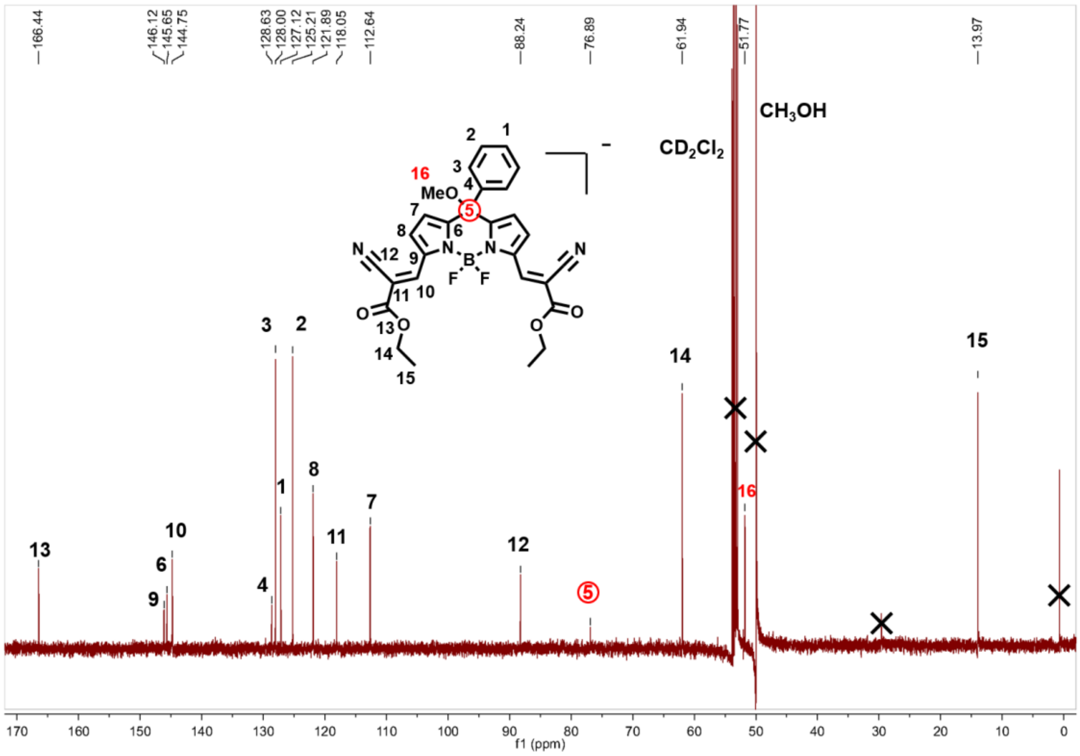

Supplementary Figure 17. ^{13}C NMR spectra (150 MHz) of **1** in CD_2Cl_2 upon addition of 80 μL CH_3OH in the presence of K_2CO_3 at room temperature.

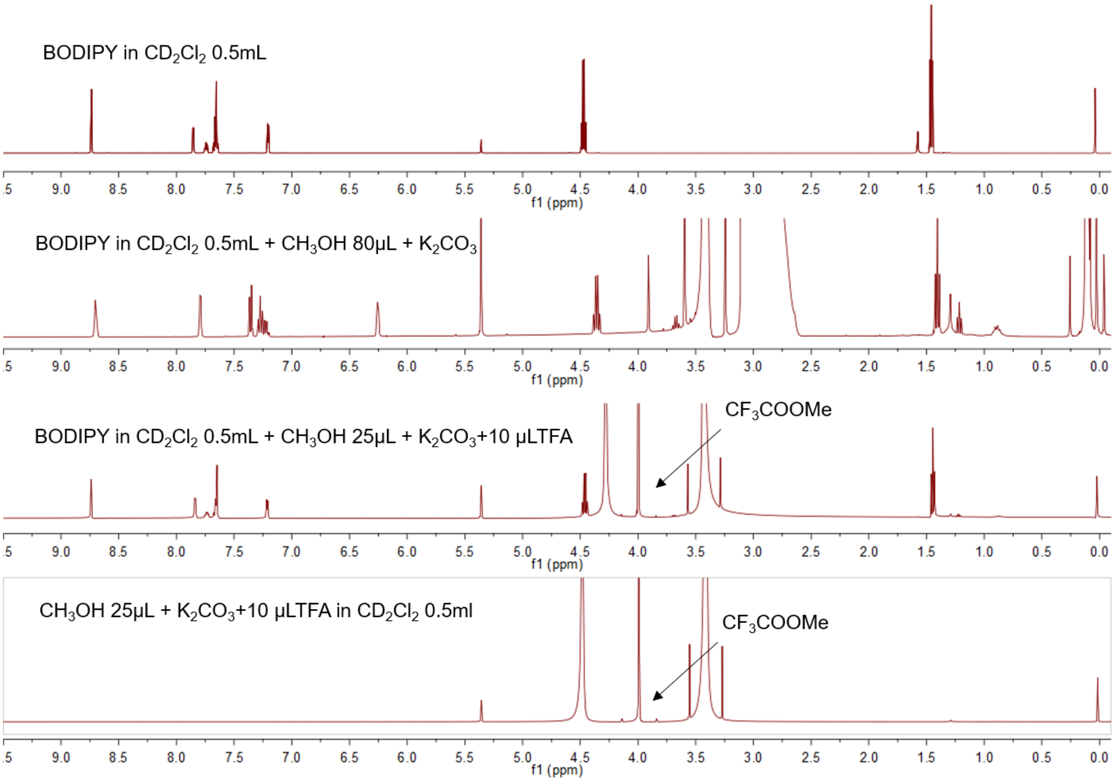

Supplementary Figure 18. Reversibility test of **1** by 1H NMR at room temperature. a)**1** in CD_2Cl_2, 298 K; b) **1** in CD_2Cl_2 upon addition of 80 μL CH_3OH in the presence of K_2CO_3, 298 K; c) **1** in CD_2Cl_2 upon addition of 80 μL CH_3OH in the presence of K_2CO_3, then add 10 μL TFA, 298K. d) 80 μL CH_3OH and 10 μL TFA in the presence of K_2CO_3 in CD_2Cl_2.

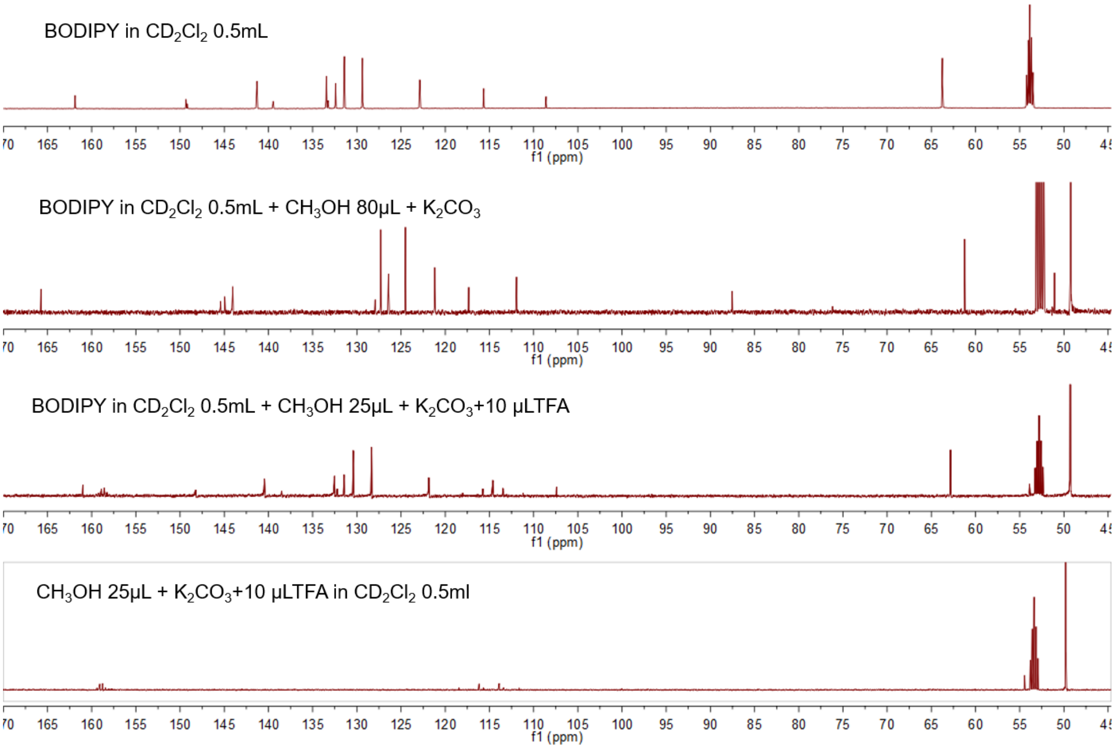

Supplementary Figure 19. Reversibility test of **1** by 13C NMR at room temperature.a)**1** in CD_2Cl_2, 298 K; b) **1** in CD_2Cl_2 upon addition of 80 μL CH_3OH in the presence of K_2CO_3, 298 K; c) **1** in CD_2Cl_2 upon addition of 80 μL CH_3OH in the presence of K_2CO_3, then add 10 μL TFA, 298K. d) 80 μL CH_3OH and 10 μL TFA in the presence of K_2CO_3 in CD_2Cl_2.

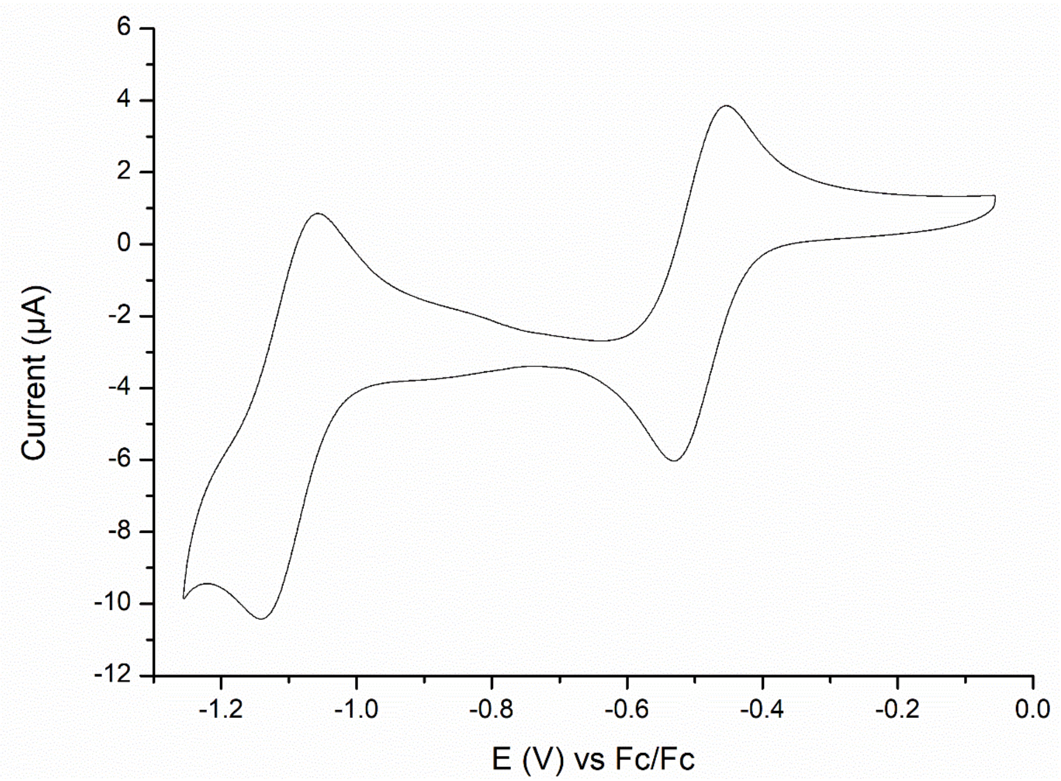

Supplementary Figure 20. Cyclic voltammogram of **1** in CH_2Cl_2 (0.5 mM, 0.1 M Bu_4NPF_6). Spectrum was measured with a glassy carbon electrode at scan rate of 50 mV/s against ferrocenium/ferrocene.

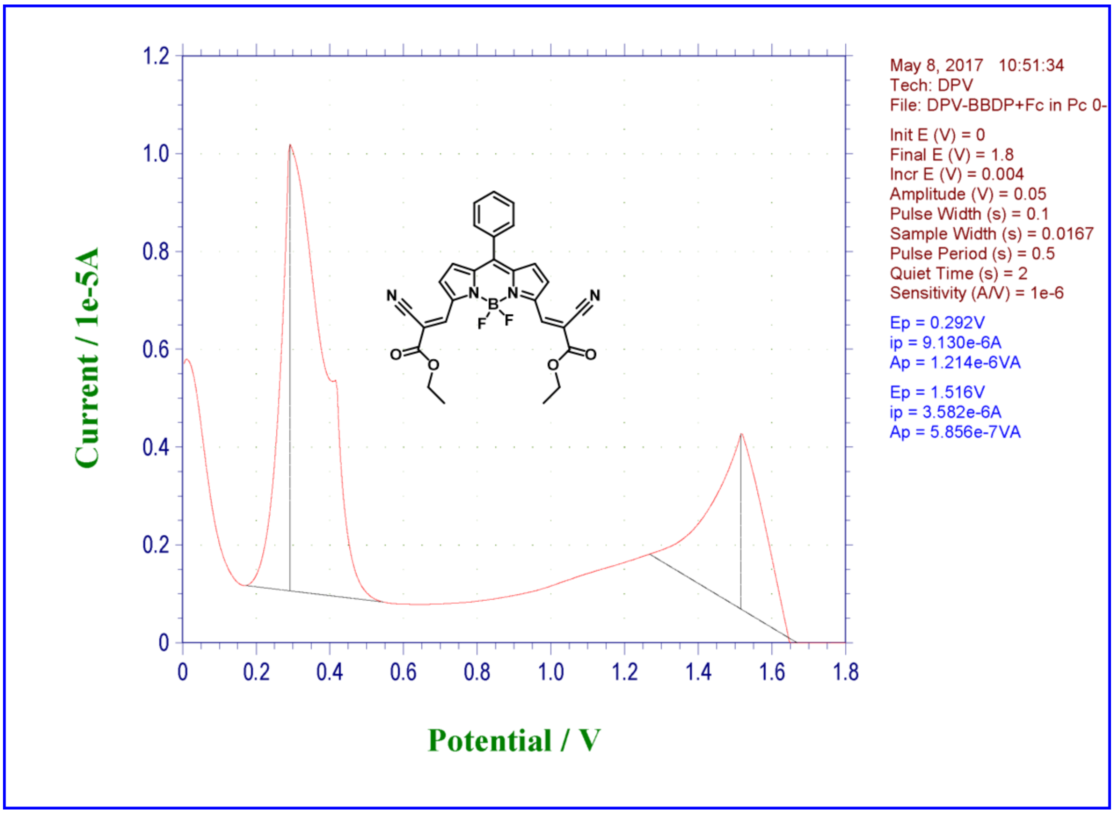

Supplementary Figure 21. Differential pulse voltammetry of **1** in CH_2Cl_2 (0.5 mM, 0.01 M Et_4NClO_4). Spectrum was measured with a glassy carbon electrode in propylene carbonate, referring to SCE. Potential range: 0 to 1.8 V; potential increment: 40 mV; pulse amplitude: 50 mV; pulse width: 50 ms; pulse period: 500 ms.

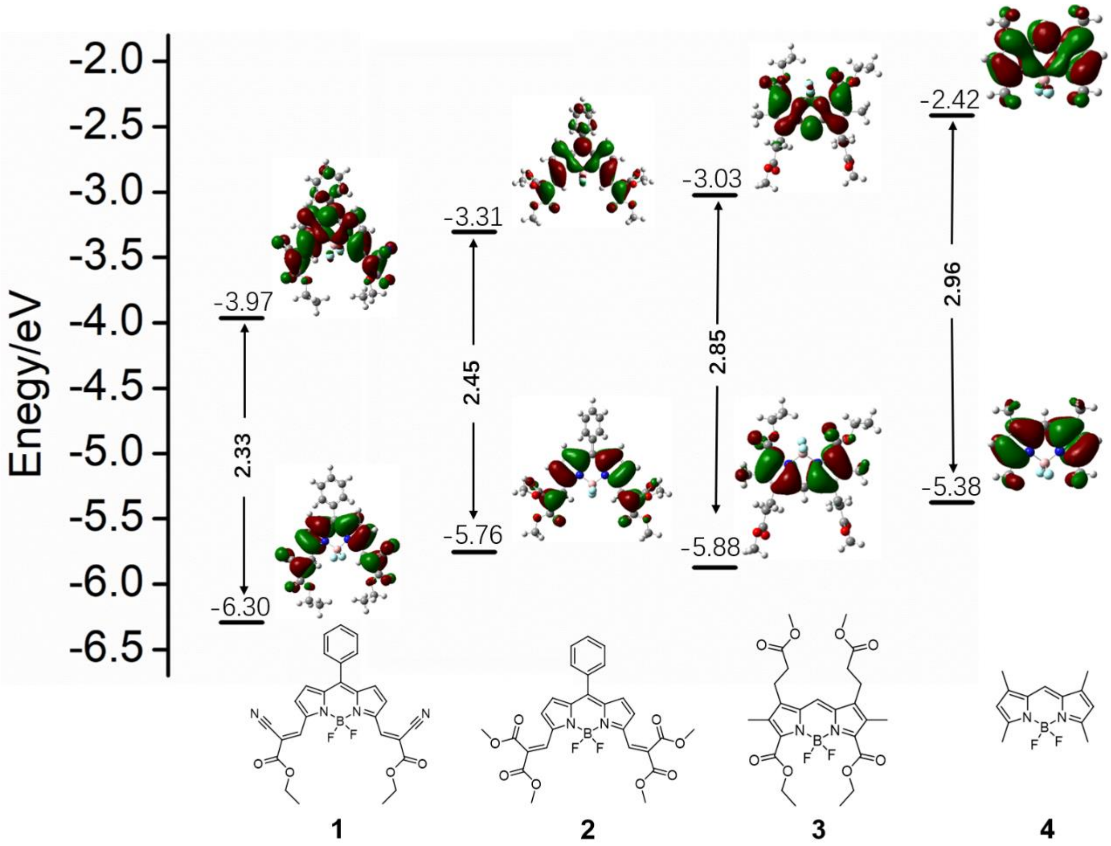

Supplementary Figure 22. Frontier Kohn–Sham MOs and their energy levels of **1**-**4**. Calculations were performed at the B3LYP/6-31G(d) level. The HOMO/LUMO energy levels were measured by DFT simulation results.

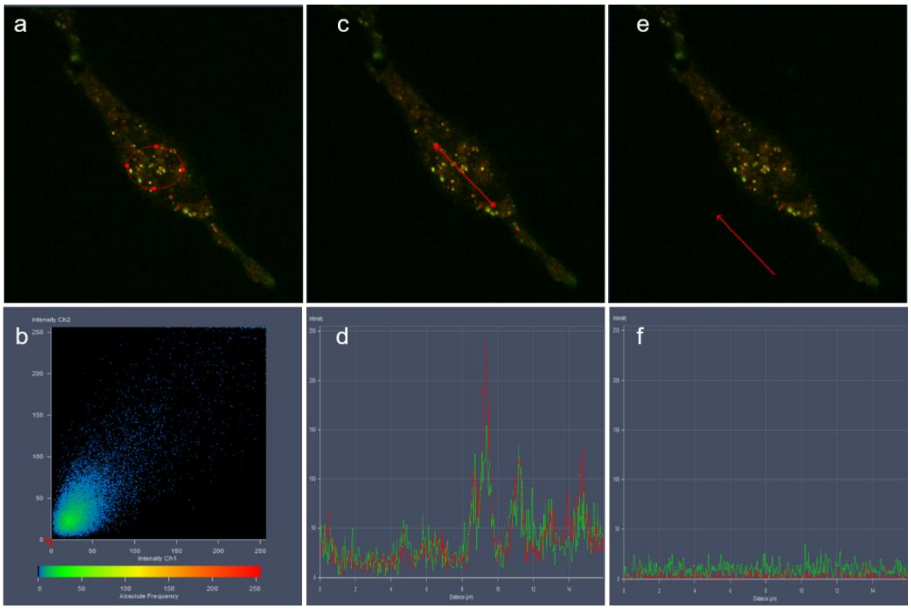

Supplementary Figure 23. Colocalization of **1** with LysoTracker Green. Region of interest (a, c, e), the Pearson intensity scatter plot (b), fluorescence intensity along the line (d, f).

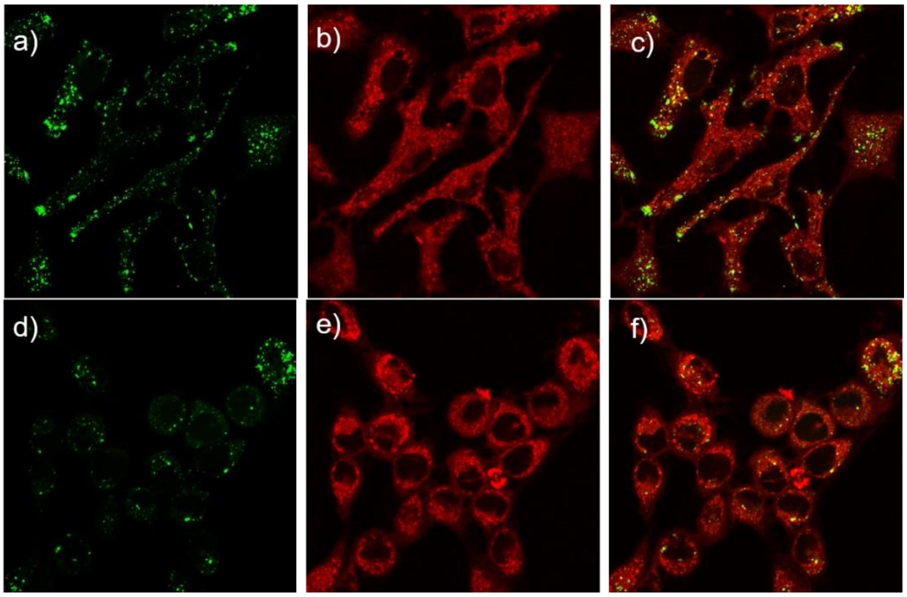

Supplementary Figure 24. Real-time fluorescence imaging of lysosomes and cytosolic acidosis simultaneously with LysoTracker Green (negative control) and 1.HeLa cells were loaded with **1** (10 μM), LysoTracker Green (0.3 μM) and dexamethasone (1 μM; 10 μM,) for 30 min and were submitted to confocal microscope observation. a) and d). Cells were excited with a 488 nm laser, and the emission was collected between 495-540 nm. b) and e). Cells were excited with a 543 nm laser, and the emission was collected between 580–730 nm. c) and f). Merged images combining a) and b), d) and e).

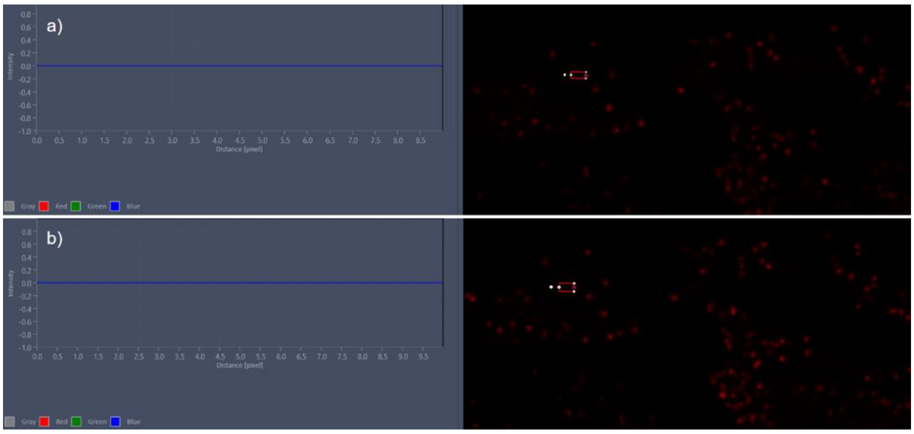

Supplementary Figure 25. The background fluorescence intensity along the straight lines with the addition of 10 μM rapamycin.a) before addition of rapamycin. b) after addition of rapamycin.

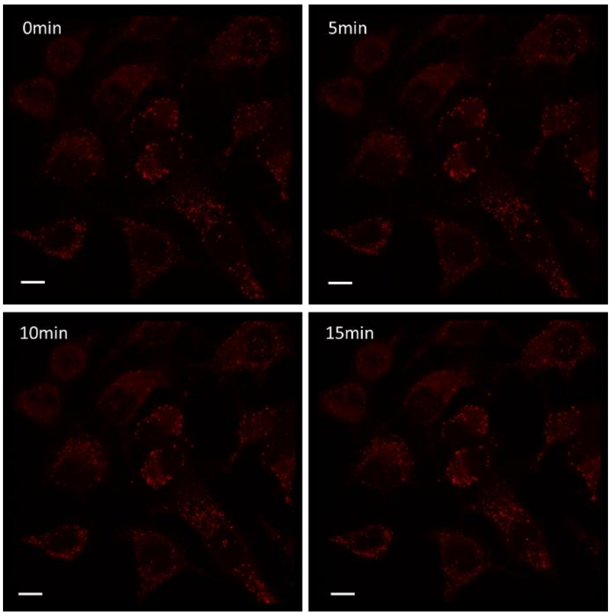

Supplementary Figure 26. Real-time fluorescence imaging of newly acidified organelles by 1 without addition of rapamycin. HeLa cells were loaded with **1** (10 μM) for 30 min and were submitted to confocal microscope observation. For each field, a series of 10-20 serial focal plane images was recorded, serial focal planes were 0.8 μm apart and were illuminated. Fluorescence images at selected time points.

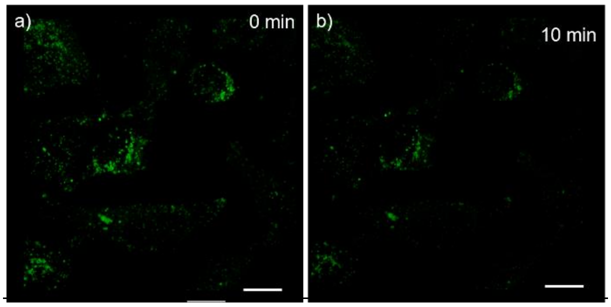

Supplementary Figure 27. Real-time fluorescence imaging of newly acidified organelles by LysoTracker Green upon addition of rapamycin. HeLa cells were loaded with LysoTracker Green (0.3 μM) for 30 min and were submitted to confocal microscope observation followed by addition of rapamycin (10 μM) under microscope. For each field, a series of 10-20 serial focal plane images was recorded, serial focal planes were 0.8 um apart and were illuminated. Fluorescence images at selected time points, scale bar 10 um.

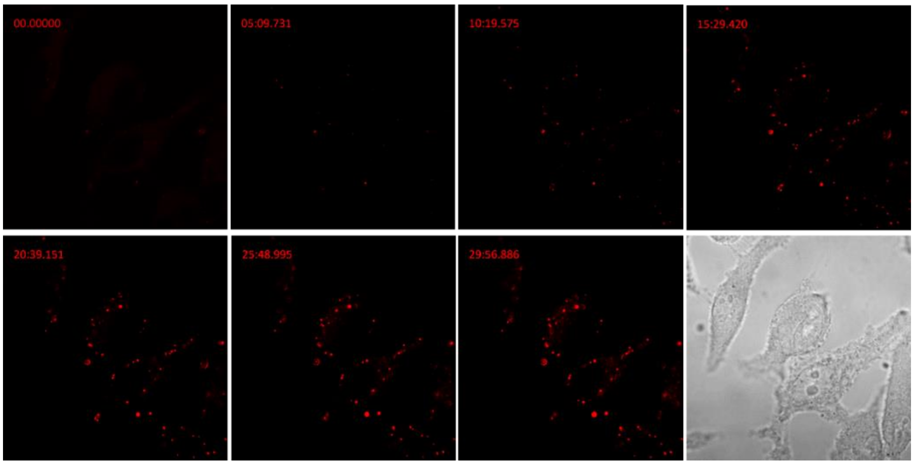

Supplementary Figure 28. Real-time monitoring of lysosome staining with **1**. Fluorescence images of living HeLa cells incubated with 10 μM **1** at 37 °C at selected time points (λ_{ex} = 543 nm, λ_{em} = 580-700 nm).

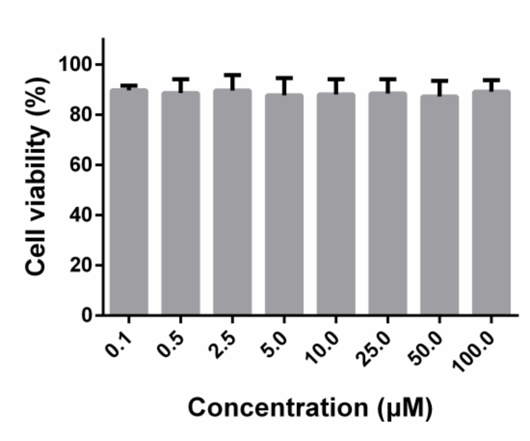

Supplementary Figure 29. Cytotoxicity tests of **1**. Cell viability after 48 hours incubated with different concentration of **1**.

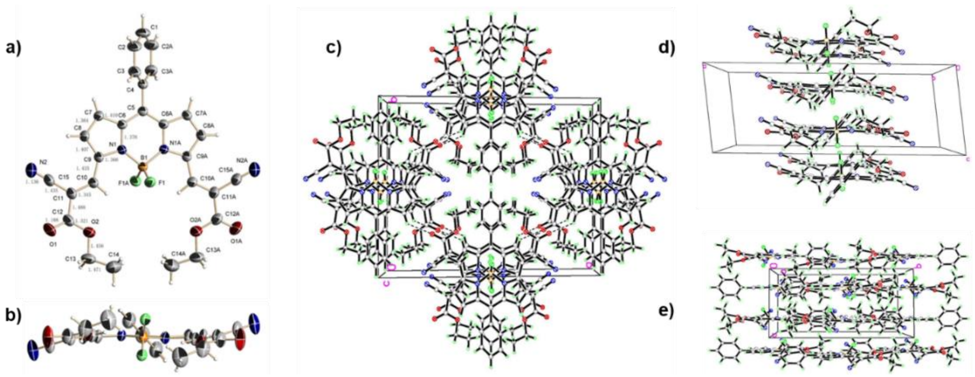

Supplementary Figure 30. Crystal structure and molecular packing of **1**. a) Perspective view. b) Side view along the B(1)-C (5) axis. c) Molecular packing in the crystal structure along the c-axis. d) Molecular packing in the crystal structure along the b-axis. e) Molecular packing in the crystal structure along the a-axis.

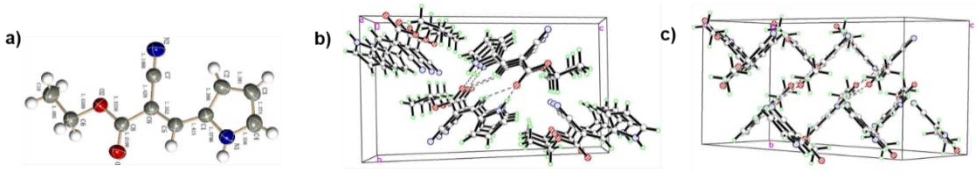

Supplementary Figure 31. Crystal structure and molecular packing of **5**. a) Molecular packing in the crystal structure of pyrrole along the a-axis. b) Molecular packing in the crystal structure of pyrrole along the c-axis. c) Molecular packing in the crystal structure of pyrrole along the b-axis.

Supplementary Table 1. Photophysical properties of compound **1** in different solvents.

	Solvent	λ_{abs}(LE) [nm]	λ_{em} [nm]	$\Delta\tilde{\nu}_{abs-em}$ [cm^{-1}]	Φ_f	τ_f [ns]
**1**	Hexane	620	626	150	0.86	4.54
	Toluene	629	643	350	0.76	5.22
	Et_2O	618	626	210	0.92	5.10
	THF	622[a]	634	300	0.87	4.42
	DCM	627	637	250	0.87	6.61
	MeCN	618	632	360	0.82	4.96
	MeOH	375	[b]	[b]	[b]	[b]

[a] ε_{max} = 110,140 M^{-1} cm^{-1}. [b] Virtually non-fluorescent.

Supplementary Table 2. Differences of chemical shifts for ^{13}C-NMR of **1** in CD_2Cl_2 and in CD_2Cl_2 containing 80 μL methanol (K_2CO_3 was added to remove any residue acid in the solvent).

No. of carbon	Chemical shift in CD_2Cl_2 / ppm	Chemical shift in CD_2Cl_2 + 80μL CH_3OH / ppm	Difference of chemical shift / ppm	Groups whose difference beyond 20 ppm
1	131.98	127.12	4.86	
2	128.93	125.21	3.72	
3	130.98	128.00	2.98	
4	132.83	128.63	4.2	
**5**	**148.75**	**76.89**	**71.86**	***meso*-canbon**
6	139.04	145.65	-6.61	
**7**	**133.02**	**112.64**	**20.38**	**pyrrole ring**
8	122.45	121.89	0.56	
9	148.90	146.12	2.78	
10	140.88	144.75	-3.87	
11	115.22	118.05	-2.83	
**12**	**108.17**	**88.24**	**19.93**	**CN**
13	161.44	166.44	-5	
14	63.32	61.94	1.38	
15	13.91	13.97	-0.06	
**16**		**51.77**		**OCH_3**

Supplementary Table 3. E_{red} (vs Fc/Fc$^+$) of compound **1**

Dye	E_1(red) vs Fc/Fc$^+$ (V)	E_2(red)* vs Fc/Fc$^+$ (V)
1	-0.50	-1.10

Supplementary Table 4. Electrochemical data and theoretical calculation results of **1-4**

	Cyclic Voltammetry					DFT Calculation		
Dye	E_{ox}/V [b]	E_{HOMO}/eV [b]	E_{red}/V [b]	E_{LUMO}/eV [c]	ΔE_{HL}/eV [d]	E_{HOMO}/eV	E_{LUMO}/eV	ΔE_{HL}/eV [d]
1	1.19	-5.97	-0.50	-4.28	1.69	-6.30	-3.97	2.33
2	-	-	-	-	-	-5.76	-3.31	2.45
3	-1.14 [a]	-5.84 [a]	-0.89 [a]	-3.68 [a]	-2.16 [a]	-5.88 (5.89) [a]	-3.03 (3.06) [a]	2.85 (2.83) [a]
4	-0.57 [a]	-5.68 [a]	-1.45 [a]	-3.34 [a]	-2.34 [a]	-5.38 (5.21) [a]	-2.42 (2.28) [a]	2.96 (2.93) [a]

[a] Reported in ref *Nat. Commun.* **9**, 362 (2018) [b] Potential for the first reduction are calculated as $E_{red} = E_{1/2}$ referred to Fc/Fc$^+$ measured by CV for reversible charge transfer, and potential for the first oxidation are calculated as $E_{ox}=E_p$ referred to Fc/Fc$^+$ tested by DPV for irreversible charge transfer; [c] The energy levels of the frontier molecular orbitals are calculated using ferrocene/ferrocenium as a reference (Fc/Fc$^+$ vs vacuum is –4.78 eV). $E_{HOMO} = -E_{ox}$–4.78 eV, $E_{LUMO} = -E_{red}$–4.78 eV; [d] The energy gap between HOMO and LUMO is calculated as $\Delta E_{H\text{-}L}=E_{LUMO}-E_{HOMO}$.

Supplementary Table 5. Crystal data and structure refinement for **1** and **5**.

	**1**	**5**
Empirical formula	$C_{27}H_{21}BF_2N_4O_4$	$C_{10}H_{10}N_2O_2$
Formula weight	514.29	190.20
Crystal size (mm^3)	0.22 x 0.19 x 0.19	0.20 x 0.20 x 0.18 mm
Crystal system	Monoclinic	Monoclinic
Space group	C 2/c	P 21/c
Z	4	4
**a** (Å)	20.885(7)	6.2750(11)
**b** (Å)	15.825(6)	9.4581(16)
**c** (Å)	7.450(3)	c = 17.273(3)
α (deg)	90	90
β (deg)	98.768(5)	108.653(5)
γ (deg)	90	90
Volume ($Å^3$)	2433.4(15)	971.3(3)
Density, calc. (mg m^{-3})	1.404	1.301
Absorption coefficient (mm^{-1})	0.105	0.093
Temperature (K)	298(2)	298(2)
F(000)	1064	400
Max. and min. transmission	0.9802 and 0.9772	0.9835 and 0.9817
Theta range for data collection (deg)	2.57 to 26.00	2.49 to 26.00
Index ranges	-17<=h<=25, -18<=k<=19, -9<=l<=8	-7<=h<=7, -11<=k<=11, -21<=l<=11
Total reflections	6103	5106
Independent reflections	2380 [R(int) = 0.0974]	1893 [R(int) = 0.0813]
Parameters	176	129
Final R indices [I>2sigma(I)]	R1 = 0.0463, wR2 = 0.1189	R1 = 0.0507, wR2 = 0.1396
R indices (all data)	R1 = 0.0766, wR2 = 0.1306	R1 = 0.0606, wR2 = 0.1489
Goodness-of-fit on F2	0.998	1.000
Largest diff. peak and hole ($eÅ^{-3}$)	0.233 and -0.414	0.306 and -0.234
