# Supplementary material for: Real-time monitoring of newly acidified organelles during autophagy enabled by reaction-based BODIPY dyes
Source: Commun Biol. 2019 Nov 28;2:442. doi: 10.1038/s42003-019-0682-1 (PMC6883057; doi:10.1038/s42003-019-0682-1)
Supplement: Supplementary file 2 — Descriptions of additional supplementary files [file 42003_2019_682_MOESM2_ESM.docx]

Supplementary Movie Legends

**Supplementary Movie 1**

Real-time imaging of Hela cells after incubated with **1** (10 μM) for 30 min upon addition of dexamethasone (10 μM).

**Supplementary Movie 2**

Real-time imaging of Hela cells after incubated with **1** (10 μM) for 30 min upon addition of rapamycin (10 μM).
